# Functional Comparison of Blood-Stage *Plasmodium falciparum* Malaria Vaccine Candidate Antigens

**DOI:** 10.3389/fimmu.2019.01254

**Published:** 2019-06-04

**Authors:** Joseph J. Illingworth, Daniel G. Alanine, Rebecca Brown, Jennifer M. Marshall, Helen E. Bartlett, Sarah E. Silk, Geneviève M. Labbé, Doris Quinkert, Jee Sun Cho, Jason P. Wendler, David J. Pattinson, Lea Barfod, Alexander D. Douglas, Michael W. Shea, Katherine E. Wright, Simone C. de Cassan, Matthew K. Higgins, Simon J. Draper

**Affiliations:** ^1^Jenner Institute, University of Oxford, Oxford, United Kingdom; ^2^Wellcome Trust Centre for Human Genetics, University of Oxford, Oxford, United Kingdom; ^3^Department of Biochemistry, University of Oxford, Oxford, United Kingdom

**Keywords:** malaria, vaccine, blood-stage malaria, antigen, merozoite, RH5, S-antigen, AARP

## Abstract

The malaria genome encodes over 5,000 proteins and many of these have also been proposed to be potential vaccine candidates, although few of these have been tested clinically. RH5 is one of the leading blood-stage *Plasmodium falciparum* malaria vaccine antigens and Phase I/II clinical trials of vaccines containing this antigen are currently underway. Its likely mechanism of action is to elicit antibodies that can neutralize merozoites by blocking their invasion of red blood cells (RBC). However, many other antigens could also elicit neutralizing antibodies against the merozoite, and most of these have never been compared directly to RH5. The objective of this study was to compare a range of blood-stage antigens to RH5, to identify any antigens that outperform or synergize with anti-RH5 antibodies. We selected 55 gene products, covering 15 candidate antigens that have been described in the literature and 40 genes selected on the basis of bioinformatics functional prediction. We were able to make 20 protein-in-adjuvant vaccines from the original selection. Of these, S-antigen and CyRPA robustly elicited antibodies with neutralizing properties. Anti-CyRPA IgG generally showed additive GIA with anti-RH5 IgG, although high levels of anti-CyRPA-specific rabbit polyclonal IgG were required to achieve 50% GIA. Our data suggest that further vaccine antigen screening efforts are required to identify a second merozoite target with similar antibody-susceptibility to RH5.

## Introduction

Development of a vaccine against *Plasmodium falciparum* malaria is a key global health priority (1). RTS,S/AS01, the leading subunit malaria vaccine, targets the pre-erythrocytic stage of the *P. falciparum* parasite's lifecycle to deliver a short interval of moderate protection against malaria (2), which then wanes to yield 7 year efficacy of < 10% (3). Blood-stage vaccines aim to target the subsequent disease-causing stage of the *Plasmodium* life cycle and have the possibility of protecting against disease severity, reducing blood-stage asexual parasitemia as well as transmission, while allowing exposure to the parasite and the development of naturally-acquired immunity.

Blood-stage vaccines can be divided into those that aim to target the infected red blood cell (iRBC), and those that target the merozoite (1). Most anti-merozoite vaccines aim to elicit antibodies that bind to proteins involved in the process of red blood cell (RBC) entry thus leading to a block in RBC invasion. This antibody functionality can be routinely tested using the standardized *in vitro* assay of growth inhibitory activity (GIA) (4). A key challenge has been to identify antigens that are both well-conserved between disparate strains of *P. falciparum*, and sufficiently susceptible to antibody neutralization in the minute or so between schizont egress and RBC re-invasion (5) in a way to meaningfully impact the *in vivo* parasite multiplication rate. *P. falciparum* reticulocyte-binding protein homolog 5 (RH5) is the only protein to-date which has been demonstrated by multiple independent research groups to possess these properties (6–9). Notably, RH5 binds basigin on the surface of RBCs, forming an interaction that is essential for productive invasion of the RBC (10).

Phase I/II clinical trials are now underway to test the safety, immunogenicity and efficacy of first-generation vaccines containing the RH5 antigen (11, 12). The outcomes of these trials will serve as an important benchmark for the field of merozoite neutralizing vaccines. When interpreting the results of these clinical trials and deciding how to prioritize further research avenues, a critical question will be whether there are additional antigens (or combinations of antigens) encoded in the *P. falciparum* genome which could deliver higher efficacy than the vaccines currently being trialed that target RH5 alone.

To address this question, we generated a list of candidate antigens for a merozoite-neutralizing vaccine and tested their ability to elicit functional antibodies, comparing to RH5 as the “gold-standard” antigen. We aimed to minimize false-negatives by adopting the following approaches. First, we used protein-based vaccine immunization which, in contrast to genetic/nucleic acid-based immunization approaches such as recombinant viral vectors (7), allows for controlled administration of a fixed dose of antigen. Second, we produced recombinant protein which, wherever possible, represented the full-length ectodomain of the *P. falciparum* protein, to minimize the possibility of producing non-conformationally relevant protein fragments as initially occurred with RH5 (13–15). Third, wherever possible, rather than use Freund's adjuvant [a strong immunopotentiator that elicits extremely high titers of antibodies but which can also disrupt the fold of even the most stable proteins (16, 17)], we used AddaVax™, a squalene-based adjuvant similar in composition to the clinically-approved adjuvant MF59®.

Testing all 5,000 proteins encoded in the *P. falciparum* genome is not feasible and so in our first panel we aimed to test all of the proteins that have been the subject of promising vaccine antigen reports in the literature. We then selected a second panel, drawn from a list of 435 proteins that have been bioinformatically predicted (18) to have a role in merozoite invasion. This was 55 proteins in all. Proteins were generated for 20 of these, and then formulated in adjuvant for immunization into mice. The post-immunization mouse antibodies were harvested and then tested for activity in the GIA assay.

## Materials and Methods

### Analysis of Polymorphism of Merozoite Proteins

The raw material for this polymorphism analysis was gathered from a range of sources [(19–22), Pf3K, 2012^1^]. We used sequence coverage of a gene as an initial indicator of vaccinological suitability. In a given dataset, sequence coverage is as defined by the number of Illumina reads that align to a given region of the *P. falciparum* clone 3D7 reference genome. Local drops in coverage can indicate divergence of an allele from the 3D7 reference, an A-T rich region (i.e., low complexity) or the presence of insertions/deletions. Therefore, genes with low sequence coverage, or spikes or drops in sequence coverage, were excluded from study. When counting single nucleotide polymorphisms (SNPs), we only considered non-synonymous SNPs with uniform sequence coverage and a prevalence of over 10%. This cut-off was chosen as a plausible indicator that a SNP is under some element of balancing selection. Polymorphism was assessed by counting the total number of non-synonymous SNPs along the gene, as opposed to measuring SNP density. This is because antigenic escape mutations may cluster around regions of a protein that are proximal in the tertiary, three-dimensional structure, but distant in the primary sequence. This simple counting analysis is unlikely to be confounded by the underlying base-rate SNP density across the genome, because the base rate of non-synonymous SNPs with prevalence of >10% is extremely low across the *P. falciparum* genome [(19–22), Pf3K, 2012^1^]. Genes with more than 4,000 bp (corresponding to 1,333 amino acids) were excluded due to the cost of producing synthetic genes longer than 4,000 bp.

### Protein Expression and Purification

Protein sequences were uploaded to the TMHMM server (23) to predict the ectodomains of transmembrane proteins of interest (for proteins with no transmembrane region, the entire soluble protein sequence was used). Synthetized genes encoding these ectodomains (GeneArt, Germany) were cloned into a modified pENTR4 plasmid. This plasmid contains an extended version of the cytomegalovirus promoter (24) downstream of an N-terminal human tissue plasminogen activator (tPA) leader sequence and cloning site encoding “GTK” (MDAMKRGLCCVLLLCGAVFVSPSQEIHARFRRGTK) and upstream of a sequence encoding a C-terminal affinity construct consisting of a biotin acceptor peptide (BAP) and either a hexa-histidine tag (“BAP-6His,” final C-terminal amino acid sequence GLNDIFEAQKIEWHEHHHHHH) or a Strep tag (“BAP-Strep,” final C-terminal amino acid sequence GLNDIFEAQKIEWHEWSHPQFEK) or simply a C-tag (25) with no biotin acceptor peptide, so final C-terminal amino acid sequence: EPEA. All genes were codon-optimized for *Homo sapiens*. The only exception to the above details was the “CyRPA (Gly KO)” construct (Figure S6), where a complete expression plasmid using a different backbone with different signal peptide and C-terminal tags was kindly gifted by the laboratory of G. Wright, who had produced this plasmid [Add gene plasmid number: 50823 (26)]. Plasmids were transiently transfected into suspension HEK293E cells (250 mL typical culture volume) using a transfection method based on that described by Durocher et al. (27). Briefly, cells were grown in Expi293 Expression Medium (Thermo Fisher Scientific, USA) in a rotary shaking, 37°C and 8% CO_2_, and passaged to 1 × 10^6^ cells/mL 24 h prior to transfection. On the day of transfection, plasmid DNA was mixed with linear 25 kDa average molecular weight polyethylenimine (PEI) at a 1:2 mass ratio (Alfa Aesar, USA), and incubated for 20 min, before being added to the HEK293E cell culture. 4 days after transfection, supernatants were harvested by spinning at 500 x*g* for 10 min and supernatants clarified with a 0.22 μm filter. Proteins were purified by single-step affinity chromatography using either a HisTrap Excel Ni-Sepharose column (GE Healthcare, USA) and eluting in 400 mM imidazole; using a C-tag with elution into 2 M MgCl_2_ (25); or dialysed extensively into phosphate-buffered saline (PBS) before application to a Streptactin Sepharose resin and elution into the supplied elution buffer (IBA, Germany). Purified proteins were exchanged into PBS using a 10 K MWCO centrifugal filter (Millipore, UK) and adjusted to 0.4 mg/mL. Proteins were resolved using 4–20% SDS-PAGE gels with reduction by 2-mercaptoethanol and visualized directly using Coomassie brilliant blue R-250 (Bio-Rad, USA), using Precision Plus protein Unstained Standards by BioRad (USA), or visualized by Western blot onto nitrocellulose membranes using streptactin-HRP conjugate (IBA, Germany) as detection reagent. PNGase treatment (NEB, USA) was performed as per manufacturer's instructions.

For the N-terminal apical asparagine-rich protein (AARP) construct in Figure S3F, a construct encoding I20 to D95 was cloned into an *Escherichia coli* expression plasmid with an N-terminal 6His tag followed by a tobacco etch virus cleavage site (amino-acid sequence ENLYFQGS). This construct was cloned into an *E. coli* expression host (BL21(DE3)Star, Thermo Fisher Scientific) and protein induced with 1 mM IPTG at 37°C for 4 h. Clarified soluble cell lysate was subject to Nickel-ion affinity purification (HisTrap™ Excel, GE Healthcare) following the manufacturer's instructions. This was followed by protease cleavage for removal of the 6His-tag and further size-exclusion chromatography, yielding a protein of apparent molecular weight 12 kDa.

### Animal Immunization

All mouse experiments used female 6 week old BALB/c mice (*n* = 4 per group). Experiments and procedures were performed in accordance with the UK Animals (Scientific Procedures) Act Project License (PPLs 30/2889 and PA7D20B85) and were approved by the University of Oxford Local Ethical Review Body. Protein (20 μg/dose) was formulated with AddaVax™ (Invivogen, France) or Abisco®-100 (Isconova, Sweden) adjuvant, and injected intramuscularly on days 0, 21, and 42. Final serum harvest was performed on day 49. Each 100 μL dose of vaccine consisted of 50 μL protein solution at 0.4 mg/mL mixed with an equal volume of AddaVax™ or Abisco®-100 adjuvant (28), and was split across both quadriceps. For Poly (I:C), we added 50 μL of Poly(I:C) adjuvant (Invivogen, USA) at 1 mg/mL to each dose of 20 μg protein and split doses across both quadriceps. In the comparative assessment of Addavax™ and Abisco®-100, in addition to the GIA negative control groups in screening, we used chicken egg ovalbumin (OVA) (Sigma Aldrich, UK). RH5 protein, used as a functional antibody immunization control was prepared as described previously (29). When RH5 viral vectors (7) were used as a functional antibody immunization control, 10^10^ viral particles (vp) of recombinant human adenovirus serotype 5 (AdHu5) were administered intramuscularly on day 1 followed by 10^8^ plaque-forming units (pfu) of recombinant modified vaccinia virus Ankara (MVA) intramuscularly on day 56, with serum harvested on day 70.

For rabbit and rat immunization, Freund's adjuvant was used to allow comparison with other studies. For S-Antigen-BAP-Strep and AARP-BAP-Strep rabbit immunization, Zika rabbits were injected intramuscularly by Biogenes (Germany). For Zika rabbits, each 400 μL dose consisted of 200 μL of protein solution at 0.5 mg/mL (100 μg dose) and 200 μL of complete Freund's adjuvant (day 1) or incomplete Freund's adjuvant (days 28 and 56). For CyRPA rabbit immunization, New Zealand White rabbits were immunized by Cambridge Research Biochemicals (UK). For these rabbits, each 400 μL dose consisted of 200 μL of protein solution at 0.5 mg/mL (100 μg dose) and 200 μL AddaVax™ adjuvant (Invivogen, France), on days 1, 28, and 56. For all rabbits the final bleed was day 63, 1 week after the final immunization. For CyRPA rat immunization, 2 Sprague-Dawley, and 2 Wistar rats were immunized intramuscularly by GenScript (Hong Kong) with 50 μg of protein formulated with complete Freund's adjuvant (day 1) and incomplete Freund's adjuvant (days 28 and 56). Rat serum was harvested on day 70 and IgG purified.

### IgG Purification

Serum from immunized mice or rabbits was diluted 2:1 in protein G binding buffer (Pierce, UK) and applied to protein G sepharose columns. After washing twice with 3x serum volume of binding buffer, IgG was eluted in 10 mL protein G elution buffer (Pierce, UK) and restored to neutral pH by the addition of 300 μL Trizma pH 9.0 (Sigma Aldrich, USA). Where RBC pre-depletion was performed, 1 μg of packed 100% hematocrit RBC per 1 μg IgG was applied to sample, incubated at room temperature (RT) with agitation for 1 h. RBC were then pelleted by centrifugation at 1,000 x*g* for 5 min and the supernatant removed.

### Immunofluorescence Assay

Cultures of 3D7 clone *P. falciparum*, synchronized to contain mainly late schizonts, were smeared onto glass slides to make thin films. Slides were fixed in 4% paraformaldehyde, blocked with 3% bovine serum albumin (BSA), and then probed with protein G purified serum IgG at 20 μg/mL. Secondary antibody was Alexa Fluor™ 488 goat anti-mouse IgG (H+L) at a dilution of 1:800 (Invitrogen, UK). Slides were mounted in ProLong Gold antifade reagent with DAPI (Life Technologies, USA). Images were acquired using a x100 oil-immersion objective and a cooled QICAM Fast 1,394 camera, each using the same exposure period.

### Assay of Growth Inhibition Activity (GIA)

These were performed to the protocol of the GIA Reference Center, NIH, USA (4). Parasites were cultured in medium consisting of RPMI-1640 supplemented with 10% heat-inactivated human serum, 5.94 g/L HEPES, 0.05 g/L hypoxanthine, and 20 mg/L gentamycin. Synchronized parasites at 0.2–0.4% parasitemia in the trophozoite stage were grown in medium containing 20% human serum and 40 mg/L gentamycin, and then mixed with test antibody and cultured for 40 h at 1% hematocrit. Test samples were prepared by taking protein G eluate (see “IgG purification”) or protein samples (see “Protein expression and purification”), and buffer exchanging three times into incomplete culture medium (lacking serum and gentamycin) using Amicon 10K MWCO centrifugal filtration devices. To deplete RBC-reactive antibodies, some samples were then incubated for 1 h with 100 μL O+ RBC at 100% hematocrit per mL of serum before centrifugation and removal of supernatant. Dialysis to exclude possibility of azide contamination was performed with Pierce MINI dialysis devices into incomplete culture medium.

Detection of parasite and inference of growth was performed using the lactate dehydrogenase method. BG98 is a rabbit anti-AMA1 purified immunoglobulin antibody pool (provided by Edmond Remarque, BPRC, Netherlands). It typically gives 80–90% GIA at 6 mg/mL and was used for quality control on assay runs.

Prediction of the GIA of an antibody mixture, GIA_A+B_, given knowledge of the GIA of individual components (GIA_A_ and GIA_B_), assuming Bliss Additivity between components, followed the equation (30):

$$\begin{array}{l} GIA_{A+B}=\left[1-\left(1-\frac{GIA_{A}}{100}\right)\times \left(1-\frac{GIA_{B}}{100}\right)\right]\times 100 \end{array}$$

### Schizont Extract Western Blot

For Figure S3E, schizont and RBC extract were prepared by taking synchronized cultures of 3D7 clone schizonts or uninfected RBC, then pelleting and resuspending in a 10x volume of Laemmli buffer containing 2-mercaptoethanol, then subjecting to three cycles of freezing and thawing. Extracts were separated on 4–20% polyacrylamide gels along with Prestained Protein Marker, Broad Range (New England Biolabs, USA) and then blotted onto nitrocellulose membrane. Membranes were probed with total IgG from immunized mice at 20 μg/mL as primary antibody, then detected with alkaline phosphatase-conjugated anti-mouse IgG secondary antibody (Jackson, USA), using p-nitrophenylphosphate (pNPP) (Sigma Aldrich, USA) dissolved in water for detection.

### Antigen-Specific Antibody Affinity Purification

CyRPA-specific rabbit IgG samples were generated in two stages. Total IgG was purified as above using protein G-sepharose. CyRPA-specific IgG was then purified from the total IgG using CyRPA-conjugated sepharose columns. These were generated using CyRPA (GlyKO)-C-tag protein and NHS-activated Sepharose 4 Fast Flow (GE Healthcare) according to the manufacturer's instructions. Total IgG was diluted 1:10 in 50 mM Na_2_HPO_4_ and applied to CyRPA-treated sepharose columns five times. After washing with 10 column volumes of 50 mM Na_2_HPO_4_ to remove the non-specific antibody, CyRPA-specific IgG was eluted with 0.1 M glycine and restored to neutral pH by addition of Trizma pH 9.0, before three rounds of buffer exchange into PBS using an Amicon 10K MWCO centrifugal filtration device.

### ELISA

96-well Maxisorp plates were coated overnight with 100 ng/well CyRPA (GlyKO)-C-tag protein (glycan sites ablated) in PBS and then blocked with 10% milk powder. Test samples and secondary antibody (anti-mouse IgG (whole molecule) produced in goat and conjugated to alkaline phosphatase, Sigma, UK) were diluted in PBS plus 0.05% Tween-20. Alkaline phosphatase activity was measured by breakdown of pNPP using optical density at 405 nm (OD_405_). Anti-OVA and anti-AARP titer was measured by defining the dilution factor required for a sample to yield a negative OD_405_, defined as 0.15 using a pre-immune negative reference. For CyRPA, a standardized ELISA was also established according to principles outlined in (31) and automated by Gen5 software (BioTek, USA). Briefly, serum from the six rabbits shown in **Figures 5**, **6** was pooled to make a standard sample whose concentration of anti-CyRPA IgG antibody was defined as 8,000 arbitrary units (AU). This sample was used to construct standard curves on assay plates. Samples were diluted such that they gave an OD_405_ lying on the linear region of the standard curve (generally between OD_405_ 0.3 and 2). The AU of the starting sample was then back-calculated based on the dilution of the sample and its position on the standard curve.

### Anti-CyRPA Antibody Quantification by Concentration-Free Calibration Analysis (CFCA)

Our approach to CFCA has been described previously (30). To generate a source of mono-biotinylated CyRPA for use on a streptavidin Biacore capture chip, HEK293 cell cultures were co-transfected with a plasmid encoding CyRPA (GlyKO) (Figure S6) and a second plasmid encoding the BirA biotin ligase, and after 4 days centrifuged and filtered to remove cell debris prior to extensive dialysis into PBS. Total IgG samples were assayed at a 1:1,000 dilution of the concentrations shown in **Figure 6C**.

### Statistics

Analysis was performed using GraphPad Prism version 5.04 (GraphPad Software Inc., USA). Tests and statistics are described in Figure Legends. To determine EC_50_ values from **Figures 5E,F**, for each individual rabbit a four-parameter sigmoidal dose-response curve was fitted to the relationship between log_10_(antibody concentration) and GIA, and then used to interpolate EC_50_s for each rabbit. To generate conversion factors for ELISA AU into μg/mL of affinity purified CyRPA-specific IgG for each individual rabbit, linear interpolation was performed between individual data points for immune rabbits and the pre-immune data point in **Figure 6A**.

## Results

### Protein-In-AddaVax™ Immunization Induces High-Titer Antibodies in BALB/c Mice

Freund's adjuvant has been widely used in the generation of high-titer polyclonal antibodies against proteins of interest. However, its suitability for the testing of potential vaccine antigens in animal models is questionable. Evidence that it can disrupt the tertiary structure of proteins (16), along with ethical issues concerning the adverse reactions associated with its use, persuaded us to consider alternatives for an antigen screening programme in mice. A previous study in our laboratory reported AbISCO®-100 as a potent, alternative preclinical adjuvant (28) and a more refined replacement for the Freund's adjuvant system. However, the promising and on-going clinical development of Matrix-M (32) (the successor formulation of AbISCO®-100), made acquiring this adjuvant difficult at the time of performing the study and so we examined instead AddaVax™, a preclinical analog of the licensed human adjuvant MF-59 which is a squalene-based oil-in-water emulsion (33, 34). Mice immunized with chicken egg ovalbumin (OVA) formulated with AbISCO®-100 and AddaVax™ produced comparable titers of anti-OVA serum IgG antibodies (Figure S1) and so we proceeded with AddaVax™ adjuvant for subsequent experiments.

### Production of a Panel of Protein-In-Adjuvant Pre-clinical Vaccines

Fifty-five proteins of interest were selected, falling into two categories:

The first category consisted of 15 proteins, described in the literature as having properties that are desirable in a merozoite-blocking vaccine. These proteins met at least one of the following criteria: (i) susceptibility to vaccine-induced antibody, *in vitro* or *in vivo*; (ii) established involvement in the process of merozoite egress from schizonts or invasion into new RBC; (iii) essential to parasite viability, generally defined by reported failures to disrupt the relevant gene; or (iv) strong association with protection from clinical malaria in sero-epidemiological studies (Table S1). Candidate antigens which have already been characterized in our laboratory such as merozoite surface protein 1 (MSP1), erythrocyte-binding antigen-175 (EBA175), RH2, and RH4 were excluded [see (7) for full list]. We have previously attempted to produce viral vectors expressing MSP2 which were not immunogenic and which were subsequently found not to express MSP2 in mammalian cells (unpublished data). We therefore also excluded this protein from the literature-selected list.

The second category contained forty proteins selected from a pool of 435 genes whose protein products are implicated bioinformatically in merozoite invasion of RBC (18). A schematic for the selection of these 40 proteins is shown in Figure S2. Of these 435 genes, 104 are predicted to encode a signal peptide (35). A signal peptide is likely to be an essential property for traffic to the parasite surface, potentially via invasion-associated organelles, and is also true of all validated targets of merozoite-neutralizing antibody including MSP1, apical membrane antigen 1 (AMA1), RH5, RH4, and EBA175. Eliminating 18 proteins longer than 1,333 amino acids (due to challenges in synthesizing genes more than 4,000 bp in length), and 16 proteins which have previously been tested in our laboratory as vaccine antigens left a total of 70.

To select the final 40 from this list of 70 we ranked proteins according to polymorphism, with the intention of selecting the least polymorphic (see Methods)—it should be noted that subsequent and ongoing consolidation of genomic data into the MalariaGen portal (www.malariagen.net) means that the numbers of SNPs, and especially the frequencies within the population, have changed since our accessing these data in mid-2012. However, using AMA1, RH5, and RH4 as benchmark proteins we found that polymorphism across the list of 70 was much lower than expected. AMA1, a protein affected by strain-specific neutralization, has 34 SNPs, making it substantially more polymorphic than any protein in the set of 70. However, even proteins likely unaffected by strain-specific neutralization such as RH5 (4 non-synonymous SNPs) and RH4 (3 non-synonymous SNPs) also ranked among the top third most polymorphic proteins in the set. In fact, 33 of 70 proteins were found to contain zero non-synonymous SNPs of >10% prevalence. Presumably, RH4, and RH5 are still under a minimal degree of immune pressure which is absent for the proteins with no SNPs. This result meant that our original plan to select the 40 least polymorphic proteins from the list of 70 would have resulted in the exclusion of RH5 and RH4, which was counter to our overall strategy of selecting proteins with a similar profile to RH5.

We therefore changed our strategy for triage by polymorphism, aiming to select proteins with evidence of immune pressure, but without so many polymorphisms as to risk strain-specific immunity, i.e., a panel of minimally polymorphic proteins. Excluding 30 of the proteins with no SNPs of >10% prevalence left 40 proteins that, on the level of SNPs, resembled RH5 far more than they resembled AMA1 (see Table S2). The final selection contained 27 proteins with 0–5 SNPs, 8 proteins with 6–15 SNPs, and 5 with 15–25 SNPs. Of the literature-selected panel of 15 proteins, 8 would also have been identified using the final bioinformatic selection algorithm (Table S1).

We also aimed to minimize the probability of producing small polypeptide fragments that do not fold correctly and hence fail to elicit a functional antibody response, as occurred with RH5 in early studies of this target (7, 13, 14). Therefore, where possible, we aimed to express the entire ectodomain of the 55 shortlisted proteins. An important decision when using mammalian expression systems is whether to retain or ablate predicted sites of N-linked glycosylation (defined as N-X-S/T, where X is any animo acid residue except proline). As various *Plasmodium* antigens have been shown to elicit functional anti-parasitic antibody responses without modification to the primary amino acid sequence after expression *in situ* from mammalian cells (36–38), we obtained constructs that encoded the amino acid sequence exactly as predicted from the 3D7 clone parasite reference genome for all but one antigen candidate [P113 being the exception, the synthetic gene was the same as that used in a previous study and already had N-linked glycan sequons ablated (39)]. For SEA-1 and CyRPA, for which particularly promising vaccine antigen characteristics have been reported in the literature (40, 41), in addition to the native amino acid sequence, we also obtained constructs encoding versions of the protein with N-linked glycan sequons ablated (comparison shown in Table S1), in order to minimize the probability of a false-negative result in screening.

**Figure 1:**
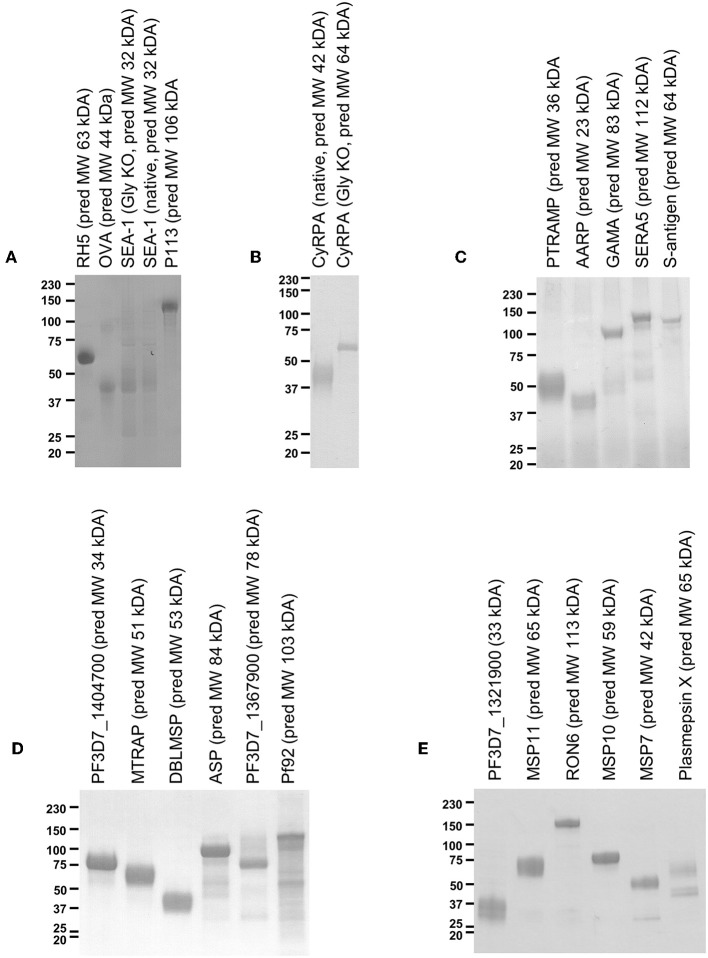
Twenty-two protein antigens representing 20 different *P. falciparum* proteins. Just prior to formulation with AddaVax™ and immunization into mice, approximately 2 μg protein was analyzed by reducing SDS-PAGE. Four immunization campaigns were run in total, with **(A,B)** combined into one campaign, and **(C–E)** each having their own campaign. **(A)** also includes analysis of “GIA positive” and “negative” control proteins, RH5, and OVA.

Mammalian expression plasmids encoding the 55 proteins of interest were successfully cloned and subsequently transfected into 500 mL cultures of HEK293E cells. Of the 55 different *P. falciparum* proteins, sufficient protein for a small scale mouse immunization campaign (approx. 300 μg, requiring expression at >0.6 μg/mL of cell culture supernatant) was obtained for 20 (Figure 1). This equates to a success rate 36.4% and is roughly in line with a previous attempt to produce a protein library using suspension HEK293 cells (42). We had significantly more success with the literature-selected panel than the bioinformatic panel (9/15 vs. 11/40, Fisher's exact test *P* = 0.0329), presumably reflecting publication bias toward proteins that can be expressed recombinantly. In a logistical regression analysis, neither protein length (OR = 0.9965, 95% CL 0.9914–1.0015) nor number of SNPs (OR = 0.8705, 95% CL 0.7125-1.0636) were predictive of expression in a statistically significant manner. Most proteins ran at a higher molecular weight than that predicted by the primary structure. Both variants of SEA-1 and CyRPA could also be expressed (Figures 1A,B). For SEA-1, no obvious difference in banding pattern was observed between the native version and the glycan-ablated version, and both constructs contained an additional band at approximately 75 kDa, which may represent the presence of a contaminant of SEA-1 dimer (Figure 1A). SEA-1 contains only two N-glycan sequons, both of which are judged unlikely to be glycosylated by the NetNGly server (http://www.cbs.dtu.dk/services/NetNGlyc/). The glycan-ablated version of CyRPA ran as a sharper band than the native version, presumably due to the presence of multiple glycoforms in the native version as CyRPA (native) contains 3 potential N-glycosylation sequons (Figure 1B and Figure S6). For many proteins, multiple smaller bands were observed, probably arising from proteolytic cleavage of the full-length construct. Given that the full-length protein was likely to be present, the presence of these smaller bands was deemed acceptable for screening purposes. Purified proteins were subsequently formulated with AddaVax™ adjuvant to produce pre-clinical protein-in-adjuvant vaccines.

### Vaccines Induced Antigen-Specific IgG With Variable Neutralizing Activity

**Figure 2:**
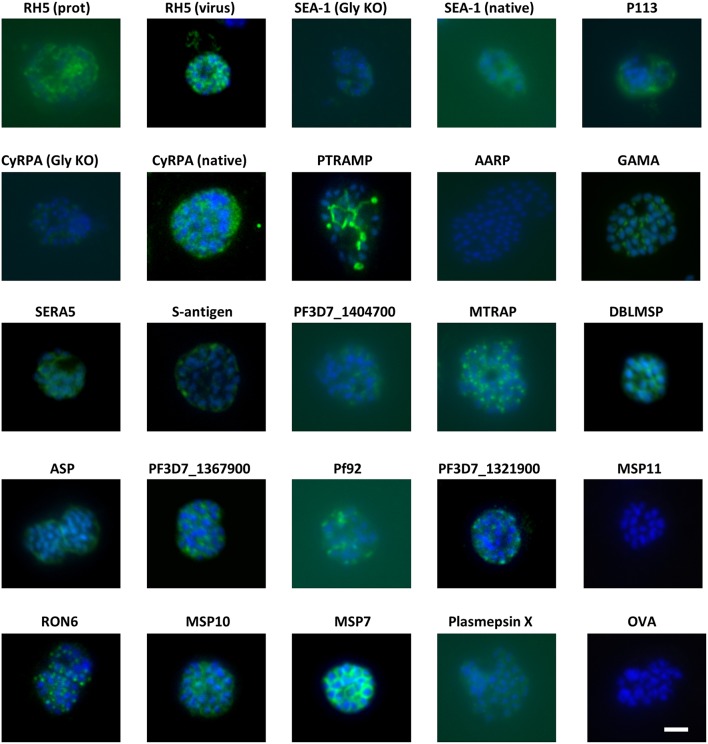
Eighteen of twenty antigens elicit antibodies that react with *P. falciparum* schizonts. Groups of 4 mice were immunized with the proteins in adjuvants as shown in Figure 1. Seven days after the final injection, serum was harvested and pooled for each group before purification of IgG using protein G. The IgG was then probed for reactivity to paraformaldehyde-fixed schizonts in an immunofluorescence assay at a total IgG concentration of 20 μg/mL. All images were captured using the same microscope settings on the same day. Scale bar = 5 μm.

Groups of 4 mice were immunized with the vaccines over several independent experiments. Each experiment contained mice immunized with RH5 (7) as a functional antibody positive control and mice immunized with OVA as a negative control. Because production of RH5 protein at the time of vaccine preparation was not yet optimized, yield was low and therefore only the first experimental group received RH5 protein-in-adjuvant vaccine (Figure 1A) while subsequent groups received RH5 viral vectors. Gels in Figure 1 show proteins immediately prior to formulation with AddaVax™ adjuvant. Following completion of the immunization phase, serum was harvested by terminal cardiac bleed. IgG was purified from the pooled sera of each group of mice and tested for binding to late schizonts by immunofluorescence (Figure 2). Total IgG from mice immunized with RH5 reacted strongly to schizonts whereas IgG from OVA-immunized mice did not. Of the remaining protein constructs tested, all but AARP-BAP-Strep and MSP11-BAP-Strep elicited IgG that reacted with schizonts. Although this experiment was not designed to assess localization, we did compare our results with previous studies to confirm similar staining patterns (Table S3). Most antibody samples showed staining consistent with previous reports, but for three proteins it was unclear whether our findings were in agreement with published work. Anti-ASP antibodies showed a punctate pattern as expected (43, 44), but additionally stained the external perimeter of the schizont. Pf92 was found to stain in a punctate manner, in contrast to a previous study using GFP-tagged Pf92 that localized to the merozoite surface (45). The GFP construct used in this study may have had non-natural trafficking, explaining the difference seen with our antibodies against native 3D7 parasite. Finally, a previous report has identified P113 as an interaction partner with RH5, and localized it to the surface of merozoites (39); however we were unable to identify any schizonts exhibiting merozoite surface staining with P113 (Figure 2), with staining more characteristic of the parasitophorous vacuole or iRBC surface, consistent with previous proteomics work (46).

**Figure 3:**
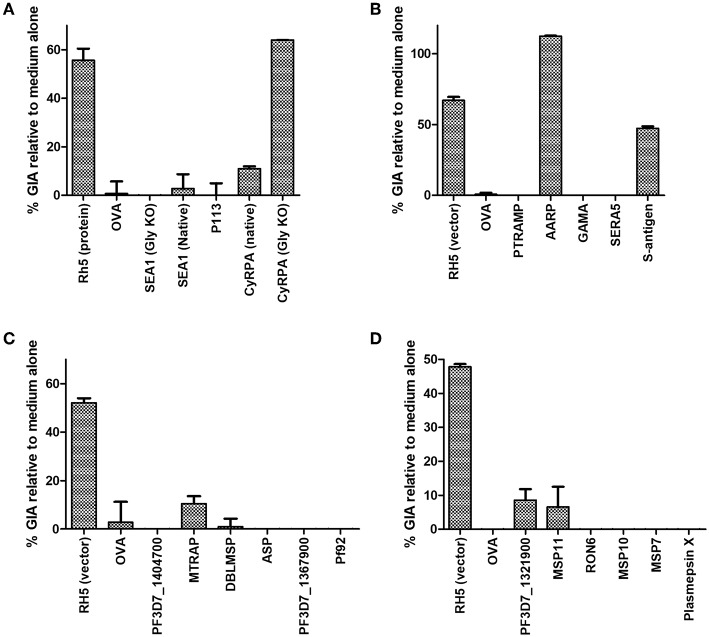
Growth inhibitory activity of antibody specificities. The protein-G purified total IgG samples tested for schizont reactivity as shown in Figure 2 were screened for activity in the standardized assay of GIA at 1 mg/mL. Each of panels **(A–D)** represents IgG from one independent mouse immunization campaign. Samples were not subjected to pre-incubation with RBC prior to GIA testing. Data is shown as mean of triplicate wells of a single GIA assay, and are representative of two independent assays.

Purified serum IgG was next screened for neutralizing activity in the *in vitro* assay of GIA at 1 mg/mL (Figures 3A–D). Anti-RH5 antibodies, whether raised using protein-in-adjuvant or virus vectors, had GIA ranging from 48 to 67%, whereas antibodies raised against OVA had < 5% GIA. In this initial screening, three vaccines appeared to have elicited functional antibodies which merited further study: CyRPA (Figure 3A), S-antigen and AARP (Figure 3B). All other IgG samples showed < 10% GIA at 1 mg/mL, which is indistinguishable from background GIA with anti-OVA antibodies.

### Investigation of Antibodies Against AARP and S-Antigen

**Figure 4:**
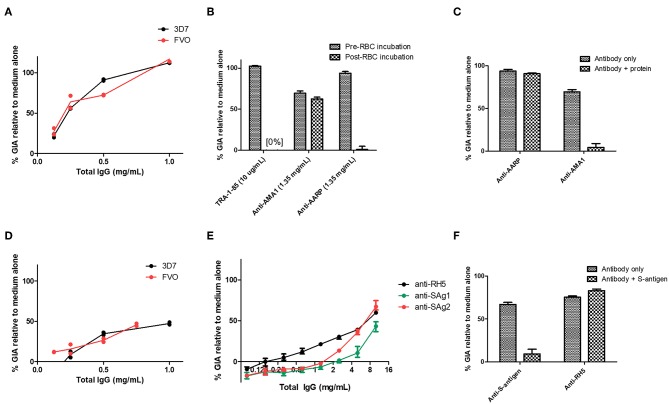
Functional activity of anti-S-antigen and anti-AARP antibodies in the assay of GIA. **(A)** Titration of the same anti-AARP-BAP-Strep sample shown in Figure 3B against the 3D7 clone and FVO strain. **(B)** Effect of RBC pre-incubation on a range of mouse antibody specificities. TRA-1-85 is a mouse anti-basigin monoclonal antibody. Other samples are total IgG from immunized mice (see Figure S3 for further details). **(C)** Pre-mixing of the relevant immunizing antigen with mouse total IgG. Final antigen concentration was 1.35 mg/mL, IgG concentration was 1 mg/mL. **(D)** Titration of the same anti-S-antigen-BAP-Strep sample shown in Figure 3B against the 3D7 clone and FVO strain. **(E)** Total IgG from two Zika rabbits immunized with S-antigen-BAP-Strep formulated with Freund's adjuvant system. Samples were subjected to pre-depletion with RBCs. **(A–D)** are each from a single GIA assay run due to sample volume constraints. **(A,D)**, lines are connected mean and points individual replicate wells (two per concentration). **(F)** Pre-mixing of S-antigen protein with pooled rabbit total IgG from **(E)**. Final S-antigen concentration was 1.5 mg/mL, total IgG concentration was 10 mg/mL. **(A–D)**, mean and range of four replicates. **(E,F)**, mean and range of three wells of a single GIA assay, representative of data from two replicate GIA assay runs.

Given the screening results above, we next sought to further analyse the antibodies against S-antigen and AARP. Murine polyclonal IgG antibodies to AARP showed high-level GIA against both 3D7 and FVO parasites (Figure 4A). However, given that the murine anti-AARP antibodies showed no discernible binding to schizonts in the IFA assay (Figure 2), their ability to neutralize parasites in the assay of GIA raised concerns about their mechanism of action and the validity of this result. We first aimed to rule out contamination of the sample with sodium azide, which is present in small amounts in the protein G binding buffer used for IgG purification. Dialysis of the small amount of remaining antibody sample showed that small molecule contamination was unlikely (Figure S3A). These experiments depleted the remaining sample of IgG from the screening experiment, however a subsequent attempt to immunize rabbits with AARP-BAP-Strep did not yield functional antibodies (Figure S3B). Therefore, a further 10 mice were immunized again exactly as for the screening study with AARP-BAP-Strep protein, and serum from pairs of mice was pooled to give five samples. Interestingly, three of the five samples showed high GIA whereas the remaining two samples showed no GIA (Figure S3C). To test whether the functional antibodies in the samples cross-reacted with the RBC surface, we pooled the five samples and assessed GIA after an RBC pre-incubation step (Figure 4B). RBC pre-treatment totally ablated all GIA of the anti-AARP antibodies in a manner comparable to the anti-basigin mAb TRA-1-85 (10) (a mAb against the RH5 receptor, used here as an anti-RBC control). Notably, these results with anti-AARP IgG contrasted with anti-AMA1 antibodies where no effect of RBC pre-incubation was seen (Figure 4B). These data suggested that the mechanism of GIA of IgG from mice immunized with AARP was through binding to a molecule on the RBC surface. Alternatively, the inhibitory antibodies raised by vaccination with this protein preparation may function by neutralizing the parasite, but also cross-react to a non-inhibitory epitope on RBCs. The negative anti-schizont IFA (Figure 2) points away from this latter conclusion.

To further interrogate this, we prepared a new batch of AARP-BAP-Strep protein and tested its ability to reverse the GIA of the murine anti-AARP IgG antibodies. GIA from the anti-AARP antibodies could not be reversed by the addition of 2.5 mg/mL of the new batch of AARP-BAP-Strep protein, whereas 2.5 mg/mL AMA1-BAP-His protein was sufficient to fully deplete the GIA activity of anti-AMA1 IgG antibodies in the assay (Figure 4C). Taken together these results did not support neutralization of parasite AARP by IgG raised in our screen as a mechanism of GIA; they also suggested that antibodies against a HEK293 cell contaminant protein in the AARP vaccine were the most likely cause of the GIA. We could also not ascertain as to why this only occurred with the AARP protein vaccine in mice (noting we failed to induce growth inhibitory IgG in rabbits immunized with the same protein), and therefore we could not exclude the possibility of murine antibodies binding to a cryptic epitope such as a transient conformer of AARP or a rare glycoform, which cross-reacted with human RBC. Of note, AARP does carry two glycosylation sequons and AARP-BAP-Strep as expressed did contain PNGase-sensitive glycans (Figure S3G). Attempts to express AARP using a construct with glycan sequons ablated were not successful in the HEK293 system (data not shown).

Given the high level of neutralization of the antibodies, and their ability to strongly inhibit the growth of two divergent *P. falciparum* strains (Figure 4A) we attempted to further characterize the mechanism of neutralization by comparing the properties of the three pools shown in Figure S3C that did have GIA with the 2 pools that did not. However, we were hampered by the relatively small sample volume that can be obtained from mouse serum and an inability to produce functional rabbit antibodies (Figure S3B). The five samples shown in Figure S3C showed no difference in anti-immunogen ELISA (Figure S3D), and no consistent difference in binding to either schizont lysate or RBC lysate as measured by Western blot (Figure S3E). Notably, previous reports of AARP as a candidate vaccine antigen have expressed a short construct of AARP from *E. coli* [amino acids I20-D107 (9, 47)]. We therefore tested a construct mirroring this published version as closely as possible. We were only able to produce a construct encoding residues I20-D95 in *E. coli* (Figure S3F). We found that when formulated in AddaVax™ and immunized into mice, antibodies elicited by this construct did not have any GIA, in contrast to AMA1 (Figure S3H).

In light of these data, we elected to not pursue this candidate antigen any further and processed to assess antibodies against the second hit in our screen—S-antigen. Here results were more promising. Murine polyclonal IgG antibodies to S-antigen showed GIA against both 3D7 clone and FVO strain parasites (Figure 4D), an effect that was reproduced against the 3D7 clone using immunized rabbit polyclonal IgG antibodies pre-depleted with RBC (Figure 4E). Importantly, mixing of purified S-antigen protein with rabbit total IgG could reverse the GIA of S-antigen antibodies, but not RH5 antibodies (Figure 4F). The antibodies isolated from mice immunized with S-antigen were probed against schizont extract by Western blot (Figure S5) and found to react to a single band of similar size to the recombinant S-antigen in Figure 1. These results suggest that S-antigen can be a target of neutralizing vaccine-induced antibodies. The ability of antibodies to neutralize 3D7 and FVO parasite was surprising because S-antigen is polymorphic (Figure S4), with 3D7 and FVO varying substantially: even among the relatively conserved 97-amino acid N-terminus of S-antigen, there are 41 sites of amino-acid divergence, with FVO and 3D7 diverging at 27 of these (Figure S4).

### Investigation of Antibodies Against CyRPA

**Figure 5:**
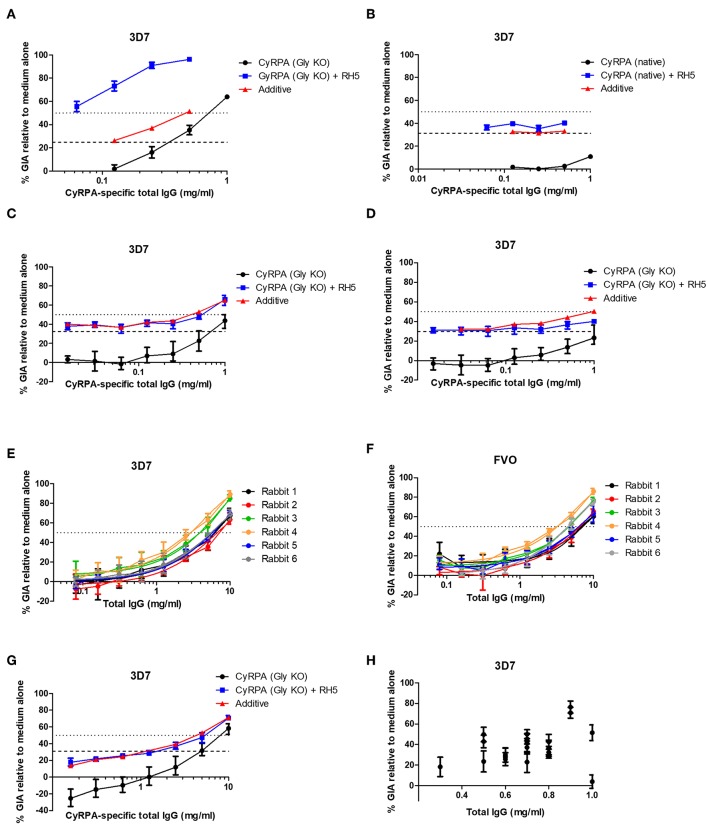
Functional activity of anti-CyRPA antibodies in the *in vitro* assay of GIA. **(A,B)** Titration of the anti-CyRPA purified IgG samples (from Figure 3A) against 3D7 clone parasites, with (blue line) and without (black line) addition of a fixed concentration (1.25 mg/mL) rabbit anti-RH5 purified IgG antibodies. Dashed line shows the activity of the anti-RH5 IgG alone tested at the single fixed concentration and red line shows the calculated activity of the mixture, assuming Bliss additivity (30). Dotted line indicates 50% GIA. **(C)** A new sample of mouse anti-CyRPA (GlyKO) IgG was generated and assessed as in **(A)**. **(D)** A new sample of mouse anti-CyRPA (Gly KO) IgG was generated using an identical immunogen to **(A,C)**, but using Poly(I:C) adjuvant for formulation. **(E,F)** Six rabbits were immunized with CyRPA (Gly KO) in AddaVax™ and their total IgG tested in a titration series for GIA against 3D7 clone and FVO strain parasites. **(G)** Pooled IgG antibodies from the six rabbits in panels **(E,F)** were tested for synergy against the 3D7 clone with anti-RH5 IgG antibodies as in **(A,B)**. **(H)** GIA of total IgG purified from 20 individual mice immunized with CyRPA (Gly KO) and AddaVax™, each tested at a different concentration as defined by IgG yield in the sample. All panels show representative results from two replicate GIA assays, except **(E)** which was repeated 3 times. Each point shows the mean of three replicate wells; error bars show the range of three replicates wells.

In our initial antigen screen, we found a striking difference in the potency of the polyclonal IgG mouse antibodies elicited to CyRPA (Gly KO) compared to CyRPA (native), both quantitatively in terms of their potency (Figure 3A) and their ability to combine with rabbit anti-RH5 IgG antibodies (7) to give a synergistic level of GIA (Figures 5A,B). Consistent with a previous report on these two antigens (41), the combination of anti-CyRPA (Gly KO) and anti-RH5 IgG here showed synergy in the assay of GIA across two replicate assays. Aside from the amino acid substitutions to remove potential sites of N-linked glycosylation (S147A, T324A, T340A, schematic in Figure S6), these constructs differed markedly. The CyRPA (GlyKO) expression plasmid was a kind gift from Gavin Wright and colleagues, who use murine Ig signal peptide, and notably include a 20.4 kDa epitope tag on CyRPA (Gly KO) derived from rat CD4 domains 3 and 4 (CD4d3+4) (Figure S6) (48). However, before investigating which, if any, of these differences in construct design had led to differing qualities of anti-CyRPA IgG, we sought to reproduce the synergy result with the CyRPA (Gly KO) vaccine and immunized new cohorts of mice and rabbits with a new batch of CyRPA (Gly KO) protein. The IgG antibodies elicited by the CyRPA (Gly KO) antigen formulated with AddaVax™ reproducibly showed GIA against 3D7 clones parasites in both mice (Figure 5C) and rabbits (Figure 5E). We also tested formulation with Poly(I:C), a fully aqueous molecular adjuvant system, and saw diminished GIA activity by comparison to AddaVax™ adjuvant in mice (Figure 5D). The rabbit anti-CyRPA antibodies also showed high-level neutralization of the FVO strain (Figure 5F). However, the synergy observed in our initial experiment (Figure 5A) was not observed in subsequent experiments using IgG purified from newly immunized mice (Figures 5C,D) or rabbits (Figure 5G); only an additive effect was observed.

Given these conflicting data, we sought to confirm the validity of the initial result showing synergy between antibodies against CyRPA (Gly KO) and RH5. It was observed in the initial experiment (Figure 5A) that the anti-CyRPA (Gly KO) purified IgG antibodies caused agglutination of the RBC in the GIA assay, and we sought to exclude this as a mechanism of GIA. We found that pre-depletion of the mouse IgG antibody sample with human RBC ablated the agglutination observed and had no effect on the GIA, either as a single specificity or its ability to synergize with anti-RH5 IgG (Figure S7A). To exclude any effect of possible anti-rat CD4d3+4 antibodies we also generated recombinant CyRPA with a C-terminal four amino acid C-tag (25), which contained the same CyRPA (Gly KO) sequence but lacked the CD4d3+4 domain, called CyRPA (Gly KO)-C-tag (see Figure S6 for construct comparisons). A vaccine containing CyRPA (GlyKO)-C-tag yielded functional antibodies comparable to the parental CyRPA (Gly KO) construct as a single specificity, however, these also showed additivity, but not synergy, with anti-RH5 IgG antibodies (Figure S7B), meaning that a role for anti-CD4 IgG cannot be excluded in the results from Figure 5A. Consequently, in a final effort to understand the mechanism of the synergy seen in Figure 5A, we immunized with CyRPA (Gly KO) and harvested IgG from individual mice, aiming to isolate monoclonal antibodies (mAbs) from mice whose serum polyclonal IgG showed synergy with anti-RH5 antibodies. IgG yields from individual mice were variable, necessitating GIA screening at a range of concentrations (Figure 5H). Although IgG from most mice showed GIA, not one of the IgG samples isolated from these 20 mice showed synergy with anti-RH5 antibodies and isolation of mAbs was not pursued (data not shown). Therefore, given the inconsistency of synergy observed in our study, we attempted to replicate as closely as possible the initial report of anti-CyRPA and anti-RH5 IgG antibody synergy, which used rat immunization with Freund's adjuvant (41). A pair of Wistar rats and a pair of Sprague Dawley rats were immunized with CyRPA (Gly KO) in Freund's adjuvant. Total IgG isolated from these rats showed 60–90% GIA at the highest concentrations tested, and some degree of additivity with anti-RH5 rabbit IgG, but no synergy was seen (Figures S7C–F). Thus, these data suggest that anti-CyRPA IgG raised in mice, rats and rabbits can elicit substantial GIA and that this is most consistently additive when combined with anti-RH5 rabbit IgG.

### Assessment of Anti-CyRPA Specific IgG Potency

Given immunization against CyRPA could reliably elicit functional antibodies, we next aimed to better assess CyRPA's suitability as a candidate vaccine antigen by quantifying its susceptibility to vaccine-induced polyclonal antibody. We and others have previously reported similar analyses for polyclonal antigen-specific IgG against RH5, AMA1, and MSP1 in different species ranging from mice through humans (1, 4, 30). Here, for rabbit anti-CyRPA antibody, the mean total IgG concentration giving 50% GIA (EC_50_) against the 3D7 clone parasite was 5.07 mg/mL (95% CI, 3.61–6.54, Figure 5E) and against the FVO strain was 5.34 mg/mL (95% CI, 3.83–6.86, Figure 5F). However, the antigen-specific IgG is typically only a small fraction of the total IgG; so we next determined the proportion of total IgG which is CyRPA-specific using two methods: (i) affinity purification of specific IgG using chromatography columns loaded with CyRPA antigen; and (ii) surface plasmon resonance analysis by calibration-free concentration analysis (CFCA) using recombinant CyRPA coated onto a capture chip. The experimental schema is shown in Figure S8.

**Figure 6:**
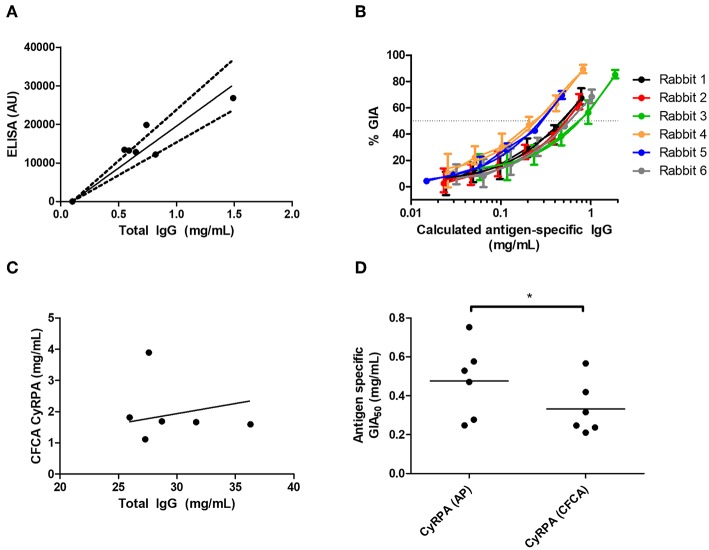
Potency of vaccine-induced anti-CyRPA antibodies from six individual rabbits. See Figure S8 for experimental schematic. A second batch of IgG was purified from the six rabbits shown in Figures 5E,F, as well as one pool of pre-immune IgG, using protein G. **(A,B)** Calculation of antigen-specific IgG concentration by affinity purification (AP) and ELISA analysis. **(A)** Total IgG from six individual rabbits was passed over a CyRPA-sepharose column and the six eluates were analyzed for total protein concentration by OD_280_, and assayed for CyRPA binding by ELISA, to generate a conversion factor for each rabbit. Points are individual rabbits, solid line is mean conversion factor (shown for information only—not used in calculations), dashed lines 95% CI (actual calculations were performed with separate conversion factors for each individual rabbit). **(B)** GIA data from Figure 5E transformed using the conversion factors determined for individual rabbits in panel A, to show CyRPA antigen-specific IgG concentration. Points are means of 3 assay well replicates, error bars show range of three replicates. **(C)** Calibration-free concentration analysis (CFCA). Total IgG was analyzed for total protein concentration by OD_280_, and anti-CyRPA antigen-specfic IgG concentration by CFCA. Pre-immune IgG gave a sub-detectable CFCA response. **(D)** Interpolated GIA EC_50_s for the six CyRPA immunized rabbits. Points are individual rabbits, bars are means.

When using the affinity-purification method, CyRPA-specific IgG was purified from total rabbit IgG using CyRPA-immobilized sepharose columns. The total protein concentration (assumed to be all antigen-specific IgG) was then analyzed by OD_280_ measurement as well as measured for anti-CyRPA responses in arbitrary units (AU) by ELISA (Figure 6A). This was used to calculate a conversion factor for ELISA AU to a specific concentration of anti-CyRPA rabbit IgG antibodies in mg/mL for each individual rabbit (for information only, mean conversion factor: CyRPA-specifc IgG concentration (μg/mL) = 0.046 (ELISA AU)−0.1; slope 95% CI 0.058–0.043). We then measured the ELISA units of the IgG stocks used to perform the GIA in Figure 5E and replotted GIA against CyRPA-specific IgG (Figure 6B).

For CFCA, total IgG from individual rabbits was analyzed to assess the concentration of anti-CyRPA antibodies in samples whose total protein concentration was measured by OD_280_ (Figure 6C). For the pre-immune rabbit IgG pool, the anti-CyRPA CFCA response was sub-detectable. For the six immune IgG samples, the mean proportion of CyRPA-specific IgG as measured by CFCA was 6.78% of the total IgG (95% CI, 2.86–10.7%). With the proportion of total IgG that was specific for CyRPA now determined for each rabbit, GIA was replotted against CyRPA-specific IgG (not shown), and the antigen-specific GIA EC_50_ values calculated with each method were compared (Figure 6D). The CyRPA-specific rabbit IgG GIA EC_50_ was calculated to be 470 μg/mL by the affinity purification method (95% CI: 270–680 μg/mL) and 330 μg/mL for the CFCA method (95% CI: 190–480 μg/mL), with the figure determined by affinity purification significantly higher than by CFCA (*P* = 0.031, Wilcoxon matched-pairs signed rank test). Both figures suggest that relatively high Ag-specific antibody concentrations are required for CyRPA polyclonal IgG mediated GIA.

## Discussion

RH5 has emerged as a leading blood-stage vaccine candidate antigen, and vaccination with this antigen appears to elicit a potent growth-inhibitory antigen-specific antibody response (1, 12). A recombinant protein vaccine called RH5.1 based on this antigen is currently in Phase I/II clinical trials (11). The objective of this study was to shed light on whether there are other antigens that could supersede or synergize with RH5 in their ability to elicit growth-inhibitory antibodies against blood-stage *P. falciparum*. This will inform the approach taken to improve on the present RH5 vaccine and/or develop next-generation vaccine formulations targeting multiple antigens to achieve even higher levels of growth inhibitory antibodies.

To meet this objective, we tested a range of merozoite proteins in as unbiased a manner as possible, testing their ability to elicit antibodies with functional GIA. RH5 was used as a “gold-standard” antigen. Fifty-five proteins of interest were selected for study: 15 were selected from the literature as having properties desirable in a vaccine antigen; while the other 40 proteins were selected on the basis of a bioinformatically predicted role in merozoite invasion (18). These 40 proteins were selected from a shortlist of 70 on the basis of having a higher number of SNPs. Of the 55 proteins selected, 20 were successfully expressed, and for 18 we were able to raise an antibody response that showed binding to fixed schizonts. While an expression success rate of 20/55 proteins is high compared to an expression library effort using a bacterial system (49), and in line with a recent study using suspension HEK293 cells (42), this leaves 35 proteins unaddressed by this study. Alternative expression systems using insect cells (29) and protozoan cells (50) may achieve a higher success rate in future studies.

When tested for function in the *in vitro* assay of GIA, three antibody specificities showed functional activity when tested alone: S-antigen; AARP and CyRPA. While our finding that S-antigen can be a target of neutralizing antibody is novel, AARP and CyRPA have been the subject of previous publications highlighting promise for these proteins as clinical candidate vaccine antigens. We therefore scrutinized the latter two antigens particularly closely, to help inform the immediate prioritization of antigens for inclusion in the next generation of clinical vaccine candidates. For S-antigen, purified IgG samples from immunized mice and rabbits (that had also been pre-depleted with RBC) were able to inhibit growth in a reproducible manner, and this effect could be reversed by pre-incubation with S-antigen protein. S-antigen has been largely unstudied since the 1980s, and was included in this screen because of statements in review articles authored by Cowman *et al*. that S-antigen is refractory to genetic ablation (51, 52). Interestingly, while most of the 14 other proteins that were initially selected based on literature reports as showing a role in merozoite invasion would also have been identified using the bioinformatics approach used to identify the other 40 proteins (18), S-antigen was not identified bioinformatically as having a role in this process. It is therefore surprising that S-antigen appears to be the target of neutralizing antibody in the GIA assay. Given the extreme variability of S-antigen between strains [Figure S4, and also (53)], the ability of antibodies raised to the 3D7 clone sequence of S-antigen to neutralize the FVO strain was particularly unexpected. Although we did not follow-up this result further, our data suggest S-antigen may warrant re-visiting in future vaccine studies, with particular emphasis on defining the neutralizing epitopes and understanding the effect of polymorphism between strains.

Different antibody samples raised against AARP did not show consistent functional GIA, and further investigation suggested the function was likely mediated through off-target binding of antibodies to the RBC. Notably, previous publications ascribing a high level of neutralization activity to anti-AARP antibodies do not describe an RBC pre-incubation step in the purification and preparation of IgG from serum (9, 47), and we were unable to reproduce these results with a similar, albeit not identical, immunogen. Our negative results for AARP in rabbits are consistent with those described in a separate, recent antigen screen (54). Although we elected to not study this antigen any further, future identification of the RBC moiety bound by the neutralizing anti-AARP IgG samples could potentially yield important information about the mechanism of invasion of RBC by merozoites.

In the case of our final “hit,” immunization with CyRPA-based constructs could reproducibly elicit antibodies from mice, rats and rabbits with invasion-blocking activity. The first mouse IgG sample we produced exhibited synergy when combined with anti-RH5 rabbit IgG antibodies across numerous replicate assays, in line with an earlier report on this antigen combination (41). However, the fact that multiple subsequent immunizations did not yield GIA with this synergistic property, including rats immunized using an almost identical protocol to that used by Reddy et al. (41), suggests that it is highly likely a result of rare antibody clones binding to specific epitopes. It could be that use of AddaVax™ adjuvant biased the antibody response toward a non-synergistic repertoire of epitopes, although a similar result was obtained when using Freund's adjuvant in rats. Although synergy between RH5 and CyRPA IgG specificities was inconsistent, the combination did consistently result in additive GIA in our hands, in agreement with some other reports. Bustamante et al. found that the combination of RH5 and CyRPA total IgG from immunized rabbits alternated between synergy and sub-additivity in the GIA assay depending on the concentration used (54), while Favuzza *et al*. appear to observe an additive interaction between RH5 and CyRPA mAbs (55). Given CyRPA, RH5 and Ripr form a complex (56, 57), it is possible that individual antibody clones that block or stabilize different aspects of complex formation could mediate either synergistic or additive interactions with anti-RH5 antibodies. However, the relationship between antibody potency and the role of the target antigen protein in merozoite invasion is far from clear: we have previously found that antibodies to RH5 interact synergistically with antibodies to RH2, RH4, and EBA175, additively with antibodies to MSP1 and sub-additively with antibodies to AMA1 (30), none of which are known to interact directly with RH5.

Further characterization of the rabbit anti-CyRPA IgG indicated that the antigen-specific GIA EC_50_ is substantially higher than that of vaccine-induced antibodies to RH5 (30) and AMA1 (58), and more akin to the levels required when targeting MSP1 (4). These data would argue against prioritization of CyRPA over RH5 for inclusion in a single-subunit blood-stage malaria vaccine. However our results are inconsistent with recent work published by Bustamante et al., who found that anti-CyRPA rabbit antibodies are at least as potent as anti-RH5 antibodies (54). Indeed, the GIA EC_50_ reported by Bustamante *et al*. for total IgG from CyRPA-immunized rabbits against 3D7 clone parasites is 0.3834 mg/mL. This is more than 10 times lower than our total IgG GIA EC_50_ of 5.07 mg/mL, and equivalent to our calculated antigen-specific GIA EC_50_. Both studies used very similar immunogens and also the same contractor for rabbit immunization (Cambridge Research Biochemicals). The differences in the GIA results could have arisen from other methodological factors, which could include GIA assay format and potentially differences in adjuvant selection and/or immunization regimen. We, and others, have noted quite marked differences in rabbit immunization studies with regard to overall endpoint titres raised against protein antigens, which may easily explain the discrepancy of the results for total IgG. Moreover, it is wholly possible the immunization protocols affected the quality of anti-CyRPA IgG induced in the rabbits, and this would suggest careful monitoring of the protein formulation and anti-CyRPA IgG quality is warranted in future studies. Nevertheless, the results in our present study are concordant with those published by Reddy et al. (41) and in line with the observation that the most potent anti-CyRPA mAbs yet described have a GIA EC_50_ of at least 250 μg/mL (55, 59–61); by contrast, the most potent anti-RH5 mAbs have a GIA EC_50_ of ~15 μg/mL (62). Taken together, it does appear that total IgG from RH5-based immunization tends to be more potent than IgG from CyRPA-based immunization. However, if the epitopes responsible for synergy between anti-RH5 and anti-CyRPA IgG could be identified and antibodies robustly elicited against them in combination, this could form the basis for a more effective bivalent next-generation blood-stage vaccine.

A final unexpected result from our screen was the failure of antibodies to both the glycan-ablated or the native version of SEA-1 to exhibit any GIA. Raj et al. found that SEA-1 antibodies are associated with protection from clinical malaria, and also showed that vaccination with PbSEA-1 can protect mice from *P. berghei* rodent malaria (40). Our results do not necessarily contradict these findings, because protection from malaria by anti-SEA-1 antibodies might well be mediated by Fc-dependent immune effector functions not measured in the GIA assay such as complement fixation and myeloid cell activation. However, our finding that anti-SEA-1 antibodies did not have GIA does conflict with Raj et al.'s finding that anti-SEA-1 serum antibodies, as well as mAbs, can block growth in *in vitro* cultures (40). It is possible that our protein was not folded correctly, or was insufficiently immunogenic when immunized into mice with AddaVax™ as an adjuvant, leading to a negative result. Fortunately Raj *et al*. produced mAbs to SEA-1 (40), and testing these in the standardized assay of GIA as operated by the International GIA Reference Laboratory should resolve this discrepancy.

With respect to the process used to select the “bioinformatically selected panel” of 40 proteins, even in retrospect it is difficult to say whether the approach taken was optimal. Of the 15 literature-selected proteins, 8 would have been identified with the bioinformatic selection algorithm. Among the 2 robust hits from the screen, CyRPA would have been selected using the bioinformatic approach whereas S-antigen would not have (Table S1). This result does suggest that there may be other invasion-blocking candidate antigens which were not identified as part of the “Merozoite invasion sub-network” by Hu et al. (18). It is difficult to say whether our decision to prioritize the moderately polymorphic proteins at the expense of non-polymorphic proteins was the right one. CyRPA and S-antigens, the two robust hits from this study, both came from the literature-selected panel. CyRPA has one SNP of >10% prevalence, which would mean that had it been in the 'bioinformatically-selected' panel it would have been brought forward whether we selected the 40 most polymorphic or the 40 least prevalent genes. S-antigen is so polymorphic that with the data available at the time it was not possible to actually count SNPs, and even with data available now a full discussion of polymorphism in S-antigen is beyond the scope of this paper. Both of these antigens elicit antibodies capable of neutralizing 3D7 and FVO, which differ markedley in their S-antigen sequences, suggesting that polymorphism is not always predictive of strain-specific neutralization.

While this study has focused on the assay of GIA, as one defined mechanistic correlate that is causative of protection against blood-stage *P. falciparum* (63) it should be recognized that alternative assays of anti-merozoite antibody efficacy do exist, including phagocytosis assays (64), the assay of antibody-dependent respiratory burst (65), and the assay of antibody-dependent cellular inhibition (66). Antibody performance in each of these assays has been shown to correlate with protection from clinical malaria in the context of naturally-acquired humoral immunity. However, there are technical challenges with using these assays for antibodies derived from different species as in this study. We selected the assay of GIA because our goal was to benchmark against antibodies to RH5, which are functional in this assay.

In summary, no antibody specificity in this study outperformed anti-RH5 antibodies in the GIA assay. Neither did any polyclonal antibody specificity robustly synergize with or enhance the GIA of anti-RH5 antibodies, although additivity with anti-CyRPA IgG was generally consistent. While we attempted to make this screen as comprehensive as possible, we were not able to express every candidate antigen in HEK293 cells, and new antigens continue to be described that were not included in our original list of proteins. Bustamante *et al*. have very recently reported that SERA9, RAMA, MSRP5, and EBA181 are targets of neutralizing antibody which potentially synergize with anti-RH5 antibodies (54). The RH5-interacting protein (Ripr) is another protein with a strong rationale for testing as a vaccine antigen, but which has not been well studied due to the difficulty of expressing it in bacterial and mammalian cell systems (26, 67). It now remains a priority to test these proteins in human compatible vaccine-delivery systems to find out if their inclusion in a multi-valent vaccine offers a realistic possibility of improving on RH5 alone.

## Ethics Statement

Animal experiments and procedures were performed in accordance with the UK Animals (Scientific Procedures) Act Project License (PPLs 30/2889 and PA7D20B85) and were approved by the University of Oxford Local Ethical Review Body.

## Author Contributions

JI: wrote the paper. JI and SD: conceived the study. JI, DA, MS, KW, AD, GL, JC, DP, and MH: produced and characterized proteins. JI, LB, DP, and SdC: performed animal immunization studies. JI, RB, JM, and DQ: performed GIA assays. SS and HB: performed affinity purification and Biacore analysis on CyRPA-specific antibodies. JI and JW: selected genes of interest.

### Conflict of Interest Statement

JI, DA, AD, KW, SC, MH, and SD are named inventors on patent applications relating to RH5 and/or other malaria vaccines, antibodies and/or immunization regimens. The remaining authors declare that the research was conducted in the absence of any commercial or financial relationships that could be construed as a potential conflict of interest.

## Footnotes

^1^Pf3K (2012). *The Pf3K Project* −*1.0 pilot data* release.
