# Supplementary material for: Functional Comparison of Blood-Stage Plasmodium falciparum Malaria Vaccine Candidate Antigens
Source: Front Immunol. 2019 Jun 4;10:1254. doi: 10.3389/fimmu.2019.01254 (PMC6558156; doi:10.3389/fimmu.2019.01254)
Supplement: Supplementary file 1 [file Data_Sheet_1.PDF]

Supplementary Material

Functional comparison of blood-stage *Plasmodium falciparum* malaria vaccine candidate antigens

Supplementary Figures

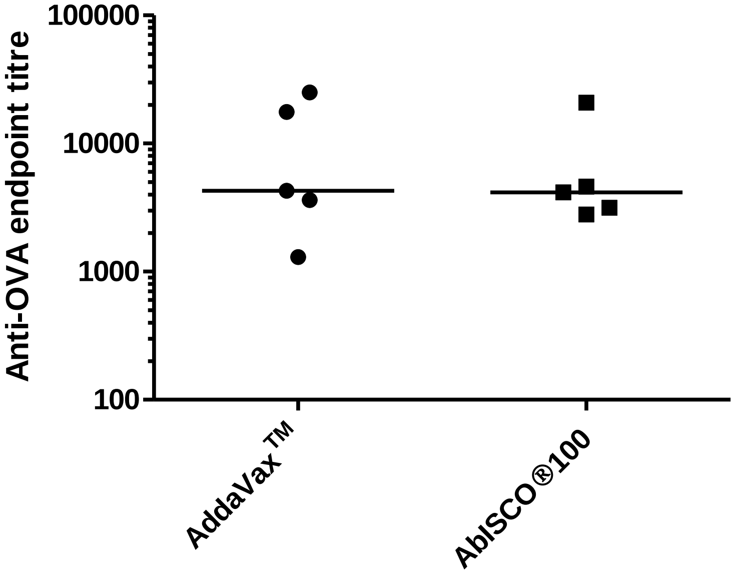

Figure S1: Comparable performance of AddaVax™ and AbISCO®-100 preclinical adjuvants. 6 week old female BALB/c mice were immunized with 20 µg hen egg ovalbumin (OVA) formulated in either AddaVax™ or AbISCO®-100 adjuvant on days 1, 28 and 56. Day 63 serum total IgG antibody responses were tested by endpoint ELISA. Data points show median and individual responses.

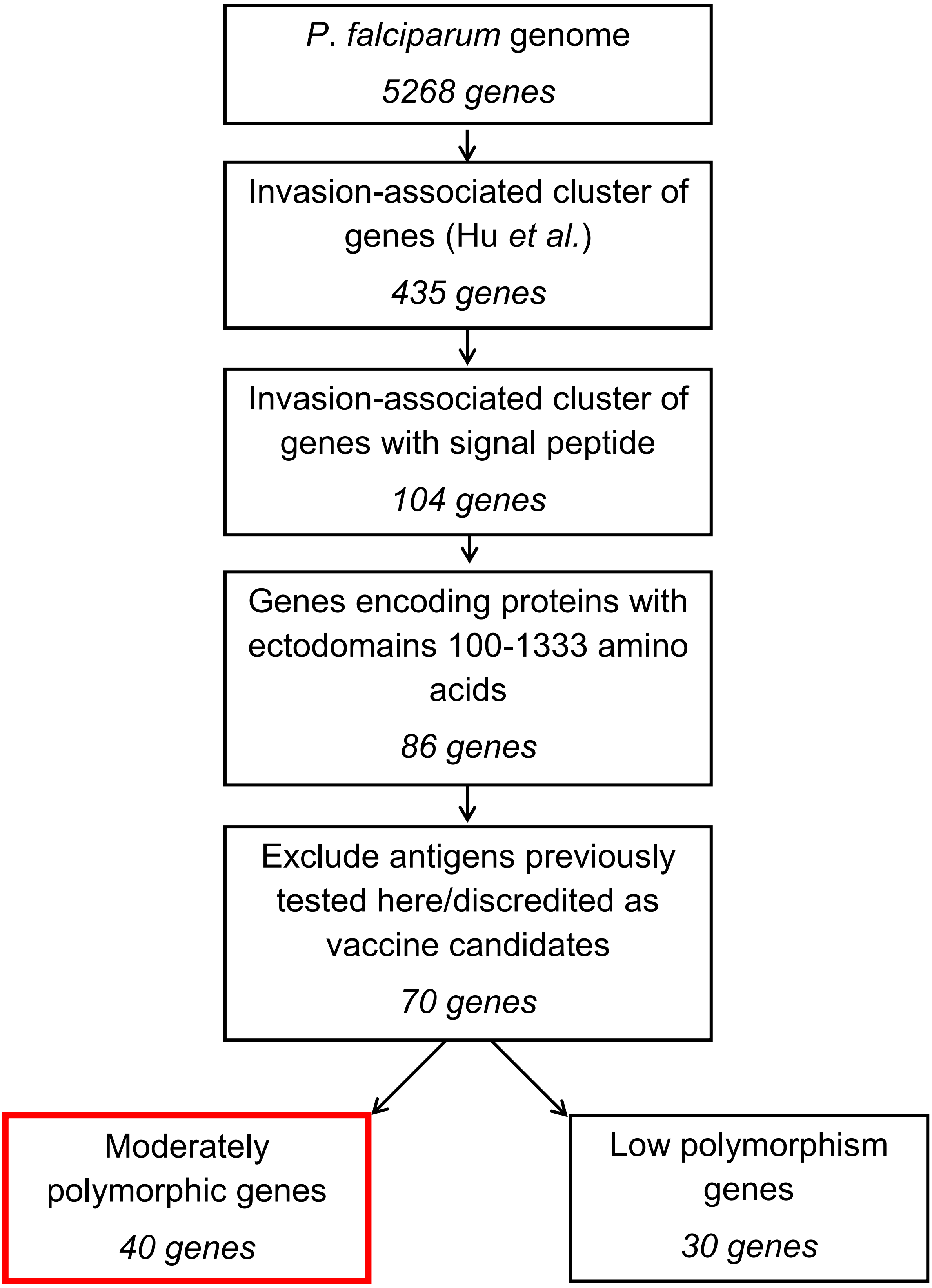

Figure S2: Selection schematic for 40 gene products implicated in RBC invasion.

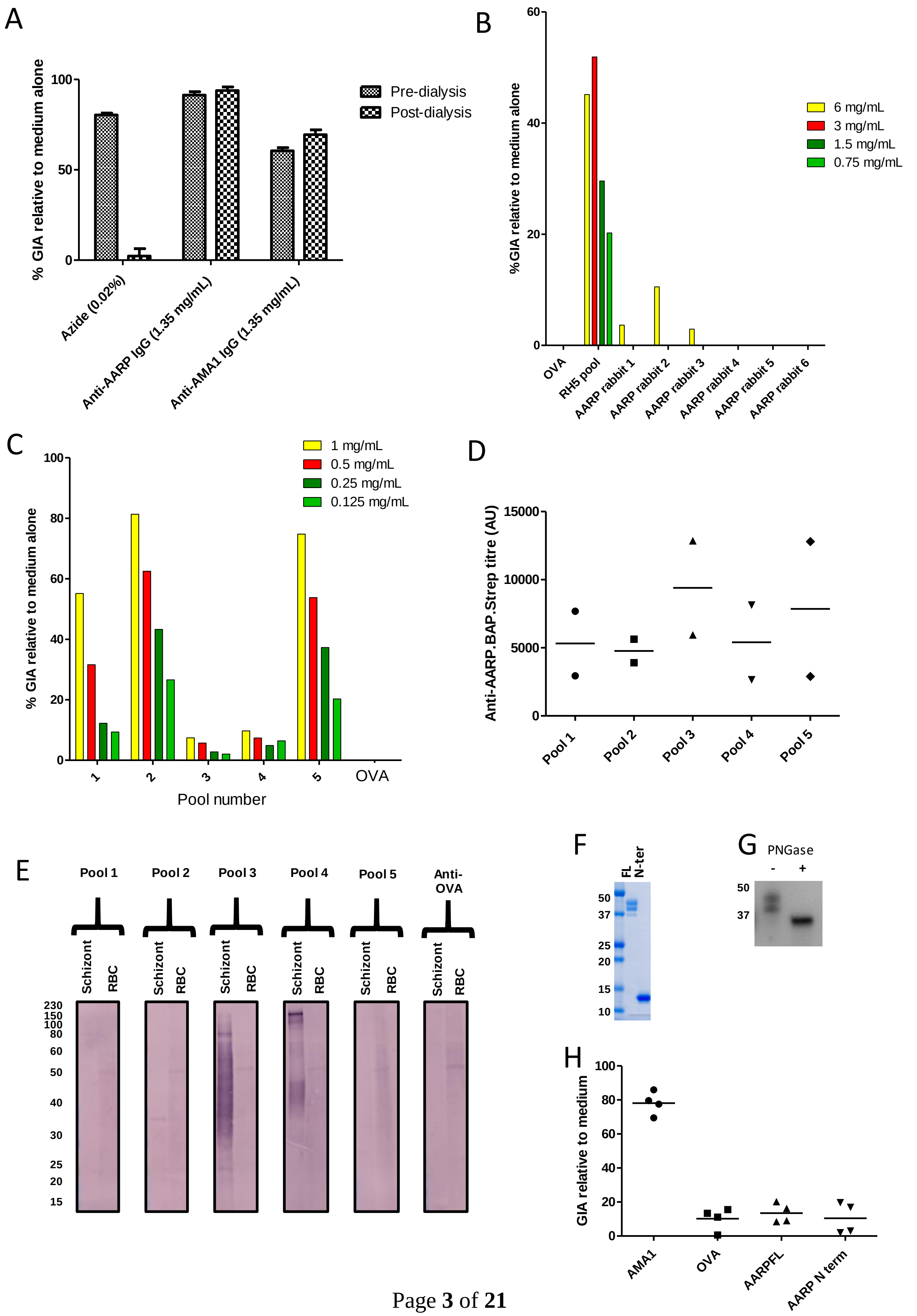
A
% GIA relative to medium alone
100
50
0
Pre-dialysis
Post-dialysis
Azide (0.02%)
Anti-AARP IgG (1.35 mg/mL)
Anti-AMA1 IgG (1.35 mg/mL)
B
%GIA relative to medium alone
60
40
20
0
6 mg/mL
3 mg/mL
1.5 mg/mL
0.75 mg/mL
OVA
RH5 pool
AARP rabbit 1
AARP rabbit 2
AARP rabbit 3
AARP rabbit 4
AARP rabbit 5
AARP rabbit 6
C
% GIA relative to medium alone
100
80
60
40
20
0
1 mg/mL
0.5 mg/mL
0.25 mg/mL
0.125 mg/mL
1
2
3
4
5
OVA
Pool number
D
Anti-AARP.BAP.Strep titre (AU)
15000
10000
5000
0
Pool 1
Pool 2
Pool 3
Pool 4
Pool 5
E
Pool 1
Pool 2
Pool 3
Pool 4
Pool 5
Anti-OVA
Schizont
RBC
230
150
100
80
60
50
40
30
25
20
15
F
FL
N-ter
50
37
25
20
15
10
G
PNGase
-
+
50
37
H
GIA relative to medium
100
80
60
40
20
0
AMA1
OVA
AARPFL
AARP N term

Figure S3: Detailed characterization of anti-AARP antibodies.

(A) Extensive dialysis of anti-AARP-BAP-Strep purified IgG was performed using 4K MWCO membranes in parallel with anti-AMA1 purified IgG and sodium azide (IgG concentration was 1.35 mg/mL). **(B)** Six rabbits were immunized with AARP-BAP-Strep protein, and the GIA of their total IgG after RBC depletion was compared with a pool of anti-RH5 purified IgG (identical results with and without RBC-depletion). Bars are means of three replicate wells. **(C-E)** Ten 6-week old female BALB/c mice were immunized with AARP-BAP-Strep protein, and 5 pools of anti-AARP-BAP-Strep IgG were made from two mice each. **(C)** GIA of the 5 pools and anti-OVA control using a dilution series of purified IgG. Bars are means of four replicate wells across two assay runs. **(D)** Anti-AARP-BAP-Strep antibody titer of the 2 mg/mL IgG pools. Points are replicate wells, bars are means. **(E)** Schizont extract and RBC extract were Western-blotted onto nitrocellulose membranes and detected with the stated antibody samples at 20 µg/µL. **(F)** SDS-PAGE analysis of an N-terminal fragment of AARP similar to that produced by Wickramarachi *et al.* was expressed in *E. coli* (purification tags cleaved off, see Methods) and compared to the full-length AARP-BAP-Strep construct expressed from mammalian cells. **(G)** Western blot of the full-length AARP-BAP-Strep construct expressed from mammalian cells, probed with Streptactin-HRP, with and without PNGase treatment. **(H)** The constructs shown in F and G were immunized into mice as per the initial screen (4 per group), serum from individual mice was harvested, IgG purified, and tested in the assay of GIA. Each data point represents GIA from the IgG of one individual mouse, lines are medians.

```
CLUSTAL multiple sequence alignment by MUSCLE (3.8)


Dd2_KOB84824.1                      MNRILSVSFYLFFLYLYIYKTYGKVKNTDQEISNIYGTNYYLRNGFLNGKNGKGNKYEDL
V1_sp|P09593.1|SANT_PLAFV           MNRILSVSFYLFFLYLYIYKTYGKVKNTDQEISNIYGTNYYLRNGFLNGKNGKGNKYEDL
SANT_PLAFN_sp|P04928.1|SANT_PLAF    MNRILSVSFYLFFLYLYIYKTYGKVKNTDQEISNIYGTNYYLRNGFLNGKNGKGNKYEDL
IGH-CR14_KNG74832.1                 MNRILSVSFYLFFLYLYIYKTYGKVKNTDHELSNIYGIKYYLRNGLSDKKNGKGQKYQDL
PFIT_1034600.1_352aa                MNRILSVSFYLFFLYLYIYKTYGKVKNTDHELSNIYGIKYYLRNGLSDKKNGKGQKYQDL
Wellcome_sp|P13821.1|SANT_PLAFW     MNRILSVSFYLFFLYLYIYKTYGKVKNTDHELSNIYGIKYYLRNGLSDKKNGKGQKYQDL
FVO_ETW18162.1                      MNRILSVSFYLFFLYLYIYKTYGKVKNTDHELSNIYGIKYYLRNGLSDKKNGKGQKYQDL
KF1916_AAA18803.1                   MNRILSVSFYLFFLYLYIYKTYGKVKNTDQEISNIYGTNYYLRNGFLNGKNGKGNKYEDL
7G8_EUR71403.1                      MNRILSVTFYLFFIYLYIYKTYGKVKNTDNELSNIYGTKYYLRSGLFNEKNYKGKKYEDL
PFHG_01341_KOB59580.1               MNRILSVTLCLFFIYLYIYKTYGKVKNTDEGLSNIYGAKYYLRSGLFNEKNGKGQKYEDL
ncbi_XP_001347627.1                 MNRILSVTLCLFFIYLYIYKTYGKVKNTDEGLSNIYGAKYYLRSGLFNEKNGKGQKYEDL
PF3D7_1035200_Sag_aa_585aa          MNRILSVTLCLFFIYLYIYKTYGKVKNTDEGLSNIYGAKYYLRSGLFNEKNGKGQKYEDL
NF54_EWC87740.1                     MNRILSVTLCLFFIYLYIYKTYGKVKNTDEGLSNIYGAKYYLRSGLFNEKNGKGQKYEDL
                                    *******:: ***:***************  :***** :****.*: : ** **:**:**

Dd2_KOB84824.1                      QEEGEGENDDEEHSNSEESDNDEENEIIVGQDGSDQE-----------------------
V1_sp|P09593.1|SANT_PLAFV           QEEGEGENDDEEHSNSEESDNDEENEIIVGQDGSDQE-----------------------
SANT_PLAFN_sp|P04928.1|SANT_PLAF    QEEGEGENDDEEHSNSEESDNDEENEIIVGQDEAPK------------------------
IGH-CR14_KNG74832.1                 EED---ENDDEEDSNSEESNNDEENKLIEGQGNQTEG-----------------------
PFIT_1034600.1_352aa                EED---ENDDEEDSNSEESNNDEENKLIEGQEGPN-------------------------
Wellcome_sp|P13821.1|SANT_PLAFW     EED---ENDDEEDSNSEESNNDEENKLIEGQEGPNSDGDKGPNSDGDKGPNSDGDKGPNS
FVO_ETW18162.1                      EED---ENDDEEDSNSEESNNDEENKLIE-------------------------------
KF1916_AAA18803.1                   QEEGEGENDDEEHSNSEESDNDEENEIIVGQDGSNEK-----------------------
7G8_EUR71403.1                      EEKREDENDDEEDLNGKISNHDEENELIEGQDSVSQ------------------------
PFHG_01341_KOB59580.1               EEEKEGENDDEEDSNSEESINDEENVLTEVQGQSE-------------------------
ncbi_XP_001347627.1                 EEEKEGENDDEEDSNSEESNNDEENELIKGQEGVEQE-----------------------
PF3D7_1035200_Sag_aa_585aa          EEEKEGENDDEEDSNSEESNNDEENELIKGQEGVEQE-----------------------
NF54_EWC87740.1                     EEEKEGENDDEEDSNSEESNNDEENELIKGQE----------------------------
                                    :*.   ******  *.: * :**** :

Dd2_KOB84824.1                      -----------------------VKVQKEQEDQEVKVQKEQEDQE--VKVQKEQEDQEVK
V1_sp|P09593.1|SANT_PLAFV           -----------------------VKVQKEQGGPGSEGPKGTGGPG--SEGPKGTGGPGSE
SANT_PLAFN_sp|P04928.1|SANT_PLAF    -----------------------SDEAEALKSDEAEALKSDEAEARKSDEAEALKSDEAE
IGH-CR14_KNG74832.1                 -----------------------TSGGKGDQTEGTSGGKGDQTEG--TSGGKGDQTEGTS
PFIT_1034600.1_352aa                -----------------------SDGDKGPNSDGDKGPNSDGDKGPNSDGDKGPNSDGDK
Wellcome_sp|P13821.1|SANT_PLAFW     DGDKGPNSDGDKGPNSDGDKGPNSDGDKGPNSDGDKGPNSDGDKGPNSDGDKGPNSDGDK
FVO_ETW18162.1                      ----------------------------------DKGPNSDGDK----------------
KF1916_AAA18803.1                   -----------------------AGSNEEAGSNEKAGSNEEAGSNEKAGSNEKAGSNEEA
7G8_EUR71403.1                      -----------------------VHGDGLGTQKGEGDGLGEGER----------------
PFHG_01341_KOB59580.1               -----------------------NHAEQGETGPGKAGEQGETGPG---------------
ncbi_XP_001347627.1                 -----------------------THGSEDEVSNGREDKVSNGGEDEVSNGGEDEVSNGRE
PF3D7_1035200_Sag_aa_585aa          -----------------------THGSEDEVSNGREDKVSNGGEDEVSNGGEDEVSNGRE
NF54_EWC87740.1                     ------------------------------------------------------------


Dd2_KOB84824.1                      VQKEQEDQEV-----KVQKEQEDQEVKVQKEQEDQEVKVQKEQEDQEVKVQKEQEDQEVK
V1_sp|P09593.1|SANT_PLAFV           GPKGTGGPGS----EGPKGTGGPGSEGPKGTGGPGSEGPKGTGGPGSEGPKGTGGPGSEG
SANT_PLAFN_sp|P04928.1|SANT_PLAF    ARKSDEAEALKSDEAEALKSDEAEARKSDEAEALKSDEAEALKSDEAEARKSDEAEARKS
IGH-CR14_KNG74832.1                 GGKGDQTEGT----SGGKGDQTEGTSGGKGDQTEGTSGGKGDQTEGTSGGKGDQTEGTSG
PFIT_1034600.1_352aa                GPNSDGDKGPNSDGDKGPNSDGDKGPNSDGDKGPNSDGDKGPNSDGDKGPNSDGDKGPNS
Wellcome_sp|P13821.1|SANT_PLAFW     GPNSDGDKGPNSDGDKGPNSDGDKGPNSDGDKGPNSDGDKGPNSDGDKGPNSDGDKGPNS
FVO_ETW18162.1                      ------------------------------------------------------------
KF1916_AAA18803.1                   GSNEEAGSNEEAGSNEEAGSNEKAGSNEKAGSNEKAGSNEEAGSNEKAGSNEEAGSNEKA
7G8_EUR71403.1                      ------------------------------------------------------------
PFHG_01341_KOB59580.1               -----------------------------------KAGEQGETGPGKAGEQGETGPGKAG
ncbi_XP_001347627.1                 DKVSNGGEDEVSNGREDKVSNGGEDEVSNGREDKVSNGGEDEVSNGREDKVSNGGEDEVS
PF3D7_1035200_Sag_aa_585aa          DKVSNGGEDEVSNGREDKVSNGGEDEVSNGREDKVSNGGEDEVSNGREDKVSNGGEDEVS
NF54_EWC87740.1                     ------------------------------------------------------------


Dd2_KOB84824.1                      V-QKEQEDQEVKVQKEQEDQEVKVQKEQEDQEV---------------------------
V1_sp|P09593.1|SANT_PLAFV           PKGTGGPGSEGPKGTGGPGSEGPKGTGGPGSEG---------------------------
SANT_PLAFN_sp|P04928.1|SANT_PLAF    D-EAEARKSDEAEARKSDEAEALKSDEAEARKS---------------------------
IGH-CR14_KNG74832.1                 GKGDQTEGTSGGKGDQTEGTSGGKGDQTEGSSG---------------------------
PFIT_1034600.1_352aa                D-GDKGPNSDGDKGPNSDGDKGPNSDGDKGPNS---------------------------
Wellcome_sp|P13821.1|SANT_PLAFW     D-GDKGPNSDGDKGPNSDGDKGPNSDGDKGPNSDGDKGPNSDGDKGPNSDGDKGPNSDGD
FVO_ETW18162.1                      ------------------------------------------------------------
KF1916_AAA18803.1                   G-SNEKAGSNEKAGSNEEAGSNEKA-----------------------------------
7G8_EUR71403.1                      ------------------------------------------------------------
PFHG_01341_KOB59580.1               EQGETGPGKAGEQGET--------------------------------------------
ncbi_XP_001347627.1                 N-GREDKVSNGREDKVSNGGEDEVSNGREDKVSNGREDKVSNGGEDEVSNGREDKVSNGG
PF3D7_1035200_Sag_aa_585aa          N-GREDKVSNGREDKVSNGGEDEVSNGREDKVSNGREDKVSNGGEDEVSNGREDKVSNGG
NF54_EWC87740.1                     ------------------------------------------------------------


Dd2_KOB84824.1                      ------------------------------------------------------------
V1_sp|P09593.1|SANT_PLAFV           ------------------------------------------------------------
SANT_PLAFN_sp|P04928.1|SANT_PLAF    ------------------------------------------------------------
IGH-CR14_KNG74832.1                 ----GKGDQTEGTSGGKGDQTEGTSGGKGDQTEG-----------------TSGGKGDQT
PFIT_1034600.1_352aa                ------------------------------------------------------------
Wellcome_sp|P13821.1|SANT_PLAFW     KGPNSDGDKGPNSDGDKGPNSDGDKGPNSDGDKGPNSDGDKGPNSDGDKGPNSDGDKGPN
FVO_ETW18162.1                      ------------------------------------------------------------
KF1916_AAA18803.1                   ------------------------------------------------------------
7G8_EUR71403.1                      ------------------------------------------------------------
PFHG_01341_KOB59580.1               ------------------------------------------------------------
ncbi_XP_001347627.1                 EDEVSNGREDKVSNGGEDEVSNGREDKVSNGREDEVSNGREDKVSNGGEDEVSNGREDKV
PF3D7_1035200_Sag_aa_585aa          EDEVSNGREDKVSNGGEDEVSNGREDKVSNGREDEVSNGREDKVSNGGEDEVSNGREDKV
NF54_EWC87740.1                     ------------------------------------------------------------
```

```
Dd2_KOB84824.1                      ------------------------------------------------------------
V1_sp|P09593.1|SANT_PLAFV           PKGTGGPGSEGPKGTGGPGSEGP----------------------------------KGT
SANT_PLAFN_sp|P04928.1|SANT_PLAF    ------------------------------------------------------------
IGH-CR14_KNG74832.1                 EGSSGGKGDQTEGTSGGKGDQTEGTSGGKGDQTEGSSGGKGDQTEG-TSGGKGDQTEGTS
PFIT_1034600.1_352aa                ------------------------------------------------------------
Wellcome_sp|P13821.1|SANT_PLAFW     SDGDKGPNSDGDKGPNSDGDKGPNSDGDKGPNSDGDKGPNSDGDKGPNSDGDKGPNSDGD
FVO_ETW18162.1                      ------------------------------------------------------------
KF1916_AAA18803.1                   ------------------------------------------------------------
7G8_EUR71403.1                      ------------------------------------------------------------
PFHG_01341_KOB59580.1               ------------------------------------------------------------
ncbi_XP_001347627.1                 SNGGEDEVSNGREDKVSNGREDKVSNGGEDEVSNGREDKVSNGGEDEVSNGREDKVSNGR
PF3D7_1035200_Sag_aa_585aa          SNGGEDEVSNGREDKVSNGREDKVSNGGEDEVSNGREDKVSNGGEDEVSNGREDKVSNGR
NF54_EWC87740.1                     ------------------------------------------------------------

Dd2_KOB84824.1                      ------------------------------------------------------------
V1_sp|P09593.1|SANT_PLAFV           GGPGSEGPKGTGGPGSEGP-----------------------------------------
SANT_PLAFN_sp|P04928.1|SANT_PLAF    ------------------------------------------------------------
IGH-CR14_KNG74832.1                 GGKGDQTEGTSGGKGDQTEGTSGGKGDQTEGSSG------------------GKGDQTEG
PFIT_1034600.1_352aa                ------------------------------------------------------------
Wellcome_sp|P13821.1|SANT_PLAFW     KGPNSDGDKGPNSDGDKGPNSDGDKGPNSDGDKGPNSDGDKGPNSDGDKGPNSDGDKGPN
FVO_ETW18162.1                      ------------------------------------------------------------
KF1916_AAA18803.1                   ------------------------------------------------------------
7G8_EUR71403.1                      ------------------------------------------------------------
PFHG_01341_KOB59580.1               ------------------------------------------------------------
ncbi_XP_001347627.1                 EDKVSNGREDEVSNGREDKVSNGGEDEVSNGREDKVSNGREDKVSNGGEDEVSNGREDKV
PF3D7_1035200_Sag_aa_585aa          EDKVSNGREDEVSNGREDKVSNGGEDEVSNGREDKVSNGREDKVSNGGEDEVSNGREDKV
NF54_EWC87740.1                     ------------------------------------------------------------

Dd2_KOB84824.1                      ----------------------KVQKEQEDQEVKVQKEQEDQEVKVQKEQEDQEVKVQK-
V1_sp|P09593.1|SANT_PLAFV           -------------------------KGTGGPGSEGPKGTGGPGSESPKGTGGPGSEGPK-
SANT_PLAFN_sp|P04928.1|SANT_PLAF    -------------------------DEAEARKSDEAEALKSDEAEARKSDEAEARKSEA-
IGH-CR14_KNG74832.1                 TSGGKGDQTEGTSGG----------KGDQTEGSSGGKGDQTEGSSGGKGDQTEGSSGGK-
PFIT_1034600.1_352aa                -------------------------DGDKGPNSDGDKGPNSDGDKGPNSDGDKGPNSDGD
Wellcome_sp|P13821.1|SANT_PLAFW     SDGDKGPNSDGDKGPNSDGDKGPNSDGDKGPNSDGDKGPNSDGDKGPNSDGDKGPNSDGD
FVO_ETW18162.1                      ------------------------------------------------------------
KF1916_AAA18803.1                   ------------------------------------------------------------
7G8_EUR71403.1                      ------------------------------------------------------------
PFHG_01341_KOB59580.1               ------------------------------------------------------------
ncbi_XP_001347627.1                 SNGGEDEVSNGREDKVSNGREDKVSNGREDKVSNGGEDEVSNGGEDEVSNGREDKVSNGG
PF3D7_1035200_Sag_aa_585aa          SNGGEDEVSNGREDKVSNGREDKVSNGREDKVSNGGEDEVSNGGEDEVSNGREDKVSNGG
NF54_EWC87740.1                     ------------------------------------------------------------

Dd2_KOB84824.1                      ------EQEDQEVKVQKEQGPKGTGGPGSEAGT------EGPKGTGG-----PGSEAGTE
V1_sp|P09593.1|SANT_PLAFV           ------GTGGPGSEGPKGTGPKGTGGPGSEAGT------EGPKGTGG-----PGSEAGTE
SANT_PLAFN_sp|P04928.1|SANT_PLAF    ----------------------GTEGPKGTGG--------------------PGSEAGTE
IGH-CR14_KNG74832.1                 ------GDQTEGSSGGKGDQTEGSSGGKGDQT-------EGSSGGKGDQTEGSSGGKGDQ
PFIT_1034600.1_352aa                KGPNSDGDKGPNSDGDKGPNSDGDKGPNSDG--------DKGPNSDGDKGPNSDGDKGPN
Wellcome_sp|P13821.1|SANT_PLAFW     KGPNSDGDKGPNSDGDKGPNSDGDKGPNSDGDKGPNSDGDKGPNSDGDKGPNSDGDKGPN
FVO_ETW18162.1                      ------------------------------------------------------------
KF1916_AAA18803.1                   -------------------GSNEEAGSNEEAGS------NEEAGSNEEAGSNEGSEAGTE
7G8_EUR71403.1                      ------------------------------------------------------------
PFHG_01341_KOB59580.1               ------------------------------------------------------------
ncbi_XP_001347627.1                 EDEVSNGREDKVSNGGEDEVSNGREDKVSNGGE------DEVSNGREDKVSNGREDEVSN
PF3D7_1035200_Sag_aa_585aa          EDEVSNGREDKVSNGGEDEVSNGREDKVSNGGE------DEVSNGREDKVSNGREDEVSN
NF54_EWC87740.1                     ------------------------------------------------------------

Dd2_KOB84824.1                      GPKGTG-------GPGSGGEHSHN------KKKSKKSIMNMLIGV
V1_sp|P09593.1|SANT_PLAFV           GPKGTG-------GPGSGGEHSHN------KKKSKKSIMNMLIGV
SANT_PLAFN_sp|P04928.1|SANT_PLAF    GPKGTG-------GPGSGGEHSHN------KKKSKKSIMNMLILM
IGH-CR14_KNG74832.1                 TEGSSG-------GDGKDGKHSHNSGNDNKNKKSKYSIINMLIGI
PFIT_1034600.1_352aa                SDGDKGPNSDGDKGPNSDGEHSRS-KNDNKKKKSKKNIINMFIGM
Wellcome_sp|P13821.1|SANT_PLAFW     SDGDKGPNSDGDKGPNSDGEHSRS-KNDNKKKKSKKNIINMFIGM
FVO_ETW18162.1                      -------------GPNSDGEHSRS-KNDNKKKKSKKNIINMFIGM
KF1916_AAA18803.1                   GPKGTG-------GPGSGGEHSHN------KKKSKKSIMNMLIGM
7G8_EUR71403.1                      -----------------DGKHSHN---------DKKNIMNMLFGM
PFHG_01341_KOB59580.1               -------------GPGKAGEQGET-------------------GP
ncbi_XP_001347627.1                 GREDKG-------GAGTDGELSHNSESHTKNKKSKNSIINMLIGM
PF3D7_1035200_Sag_aa_585aa          GREDKG-------GAGTDGELSHNSESHTKNKKSKNSIINMLIGM
NF54_EWC87740.1                     -------------------ELSHNSESHTKNKKSKNSIINMLIGM
                                                       : . .
```

Figure S4: Sequence alignment of predicted S-antigen protein. 3D7 S-antigen protein sequence (accession code: XP_001347627.1) was used to search within taxid:5833 (*Plasmodium falciparum*) of non-redundant protein sequences database using protein=protein BLAST (Blastp). A number of sequences from different regions were aligned using Multiple Sequence Comparison by Log-Expectation (MUSCLE) (Edgar, 2004). Blue boxes highlight the amino-acid residues in FVO and 3D7 at the sites where any strains diverge.

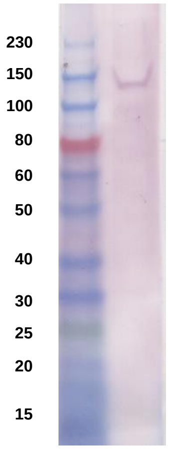

Figure S5: Selectivity of mouse anti-S-antigen antibodies.

Schizont extract was probed with antibodies from mice immunized with S-antigen. Ladder is NEB ColorPlus (Cat# P7711, now discontinued).

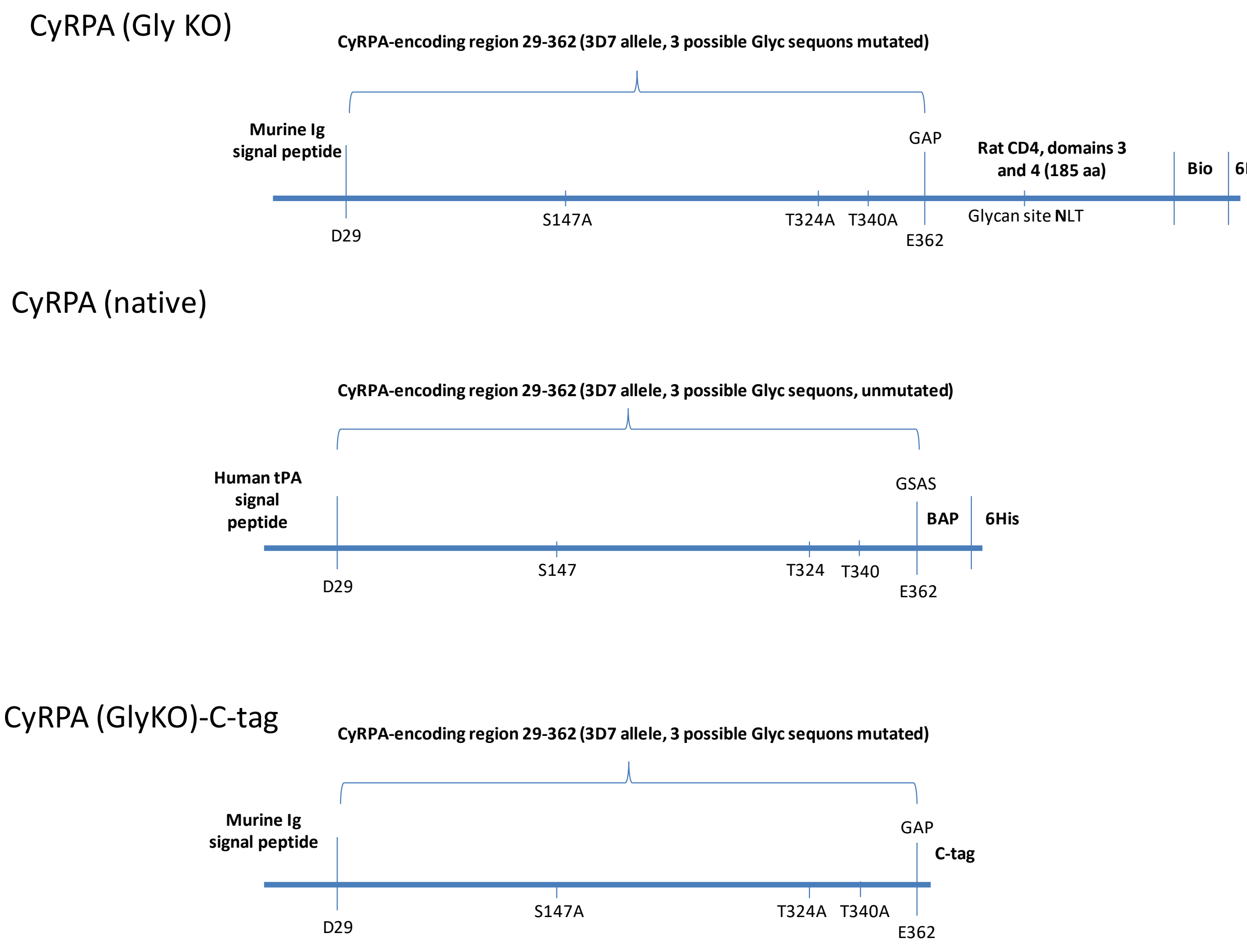

Figure S6: Schematic of CyRPA (Gly KO), CyRPA (Native), and CyRPA-C-tag.

GAP and GSAS are amino acids (FASTA format) encoded by the restriction sites between the antigen-encoding region and any C-terminal tags. Glycan sequons were any sequence encoding N-X-S/T where X was any amino acid except proline. Bio is a biotin acceptor peptide with an alternative sequence to that regularly used in our laboratory (Bushell et al., 2008).

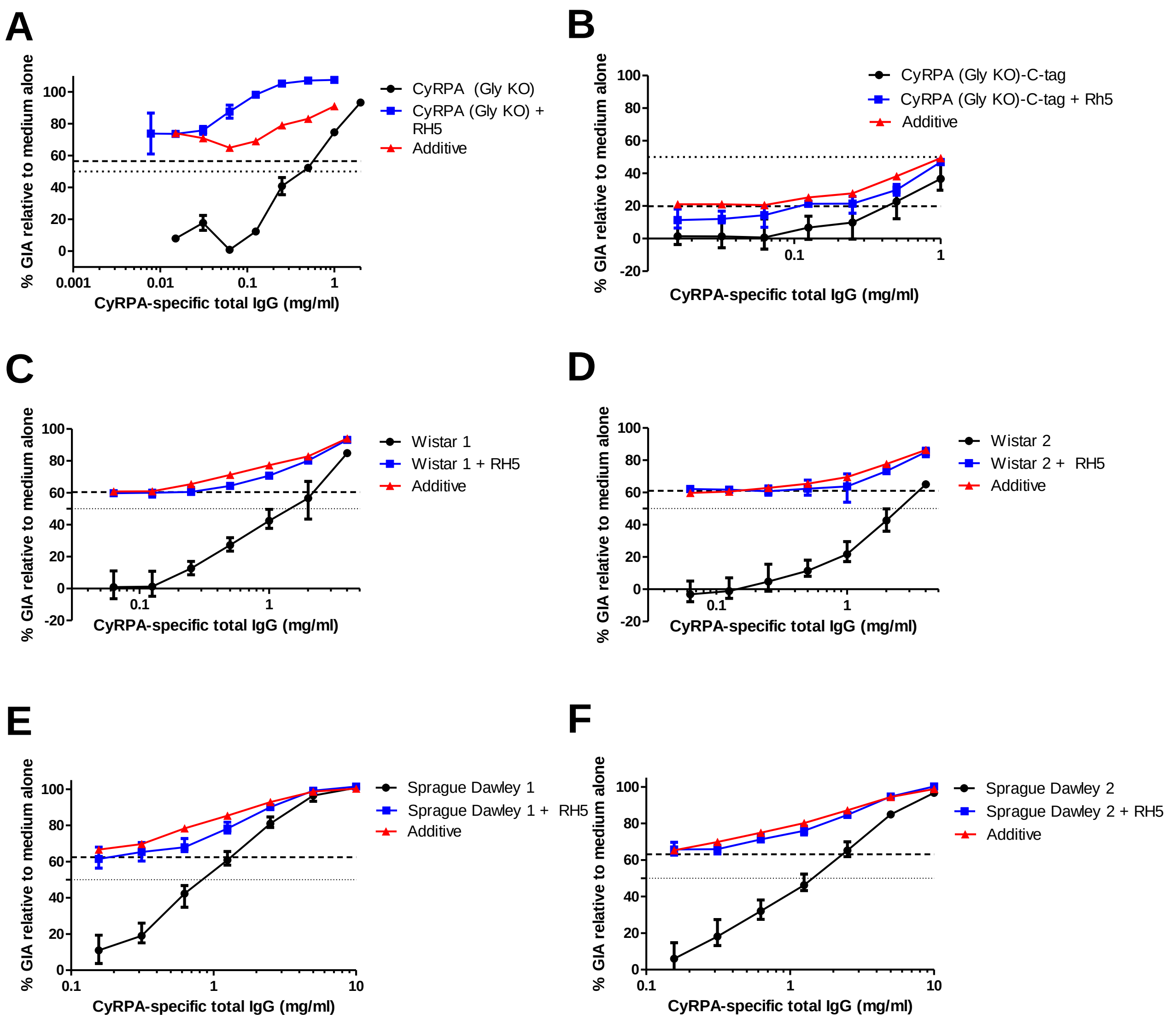

Figure S7: Growth inhibitory activity of anti-CyRPA antibodies.

Titration of anti-CyRPA purified IgG samples against 3D7 clone parasites, with (blue line) and without (black line) addition of a fixed concentration (1.25 mg/mL) rabbit anti-RH5 purified IgG antibodies. Dashed line shows the activity of the anti-RH5 IgG alone tested at the single fixed concentration and red line shows the calculated activity of the mixture, assuming Bliss additivity (Williams et al., 2012). Dotted line indicates 50 % GIA. **(A)** The sample used in Figure 5A was pre-incubated with RBC prior to performing the GIA assay. **(B)** CyRPA (GlyKO)-C-tag (with no CD4d3+4 tag) was immunized into mice and total IgG assessed for GIA. **(C-F)** Four rats, two Wistar and two Sprague Dawley, were immunized with CyRPA (KO) formulated with Freund's adjuvant. Total IgG was assessed in the GIA assay. Results are representative of two replicate GIA assays. All assays performed with 3D7 clone parasites. Each point shows the mean of three replicate wells; error bars show the range of three replicates wells.

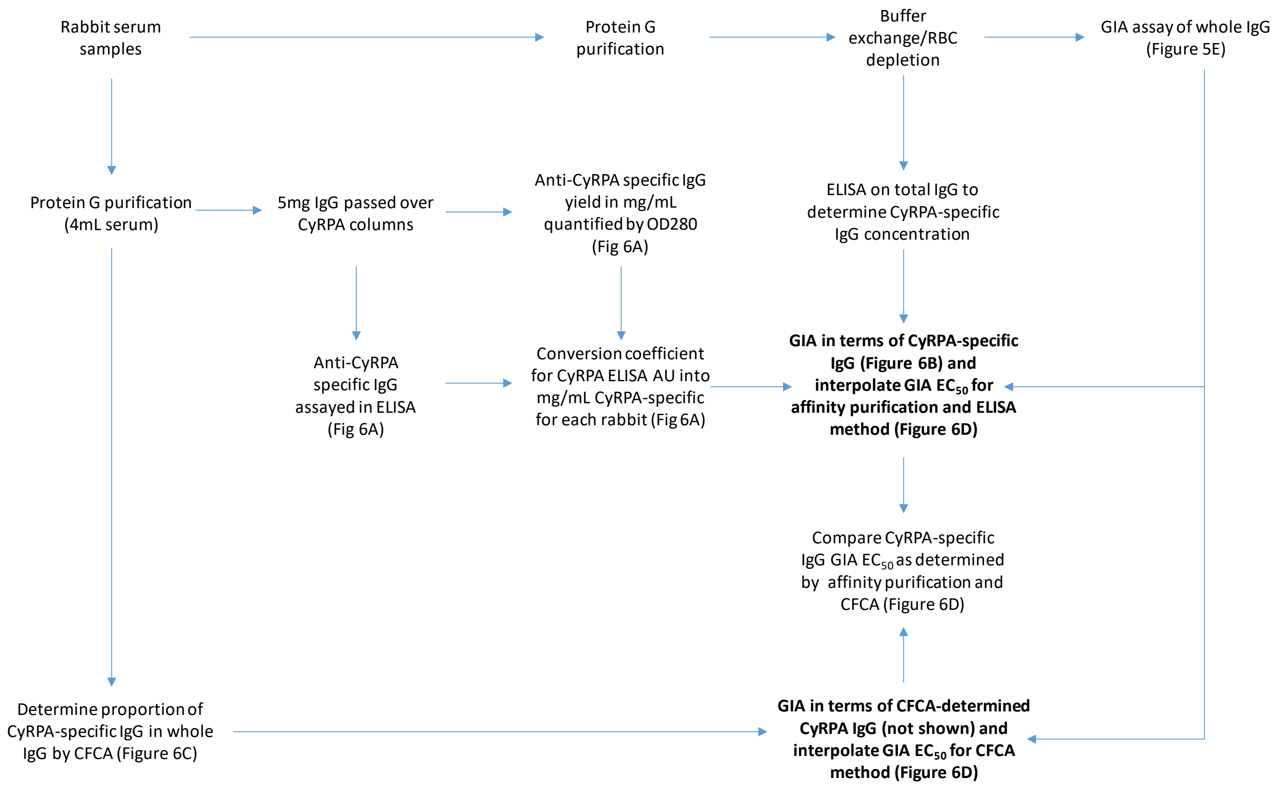

Figure S8: Schematic for determination of CyRPA-specific IgG GIA_{50} by affinity purification and calibration-free concentration analysis (CFCA).

Supplementary Tables

Table S1: Literature-selected proteins (N=15)

Gene	Protein	Construct[a]	SNPs	Sufficient protein obtained?	C-terminal tag(s)	Rationale for inclusion	Meet bioinformatic selection criteria?
PF3D7_1420700	P113	Y23—K942 (919, L969)	1	Yes	C-tag	Role in merozoite invasion (Galaway et al., 2017)	No: not in Hu et al., (2010) list of 435 invasion-associated genes
PF3D7_1218000	PTRAMP (thrombospondin-related apical membrane protein)	C28—K309 (281, D352)	0	Yes	BAP-Strep	Homologue cannot be knocked out in *P. berghei* (Thompson et al., 2004)	No: meets all criteria except too few SNPs
PF3D7_0423400	AARPdTM (apical asparagine rich protein, ectodomain only)	K18—P191 (173, P217)	1	Yes	BAP-Strep	Susceptible to vaccine-induced antibody (Pandey et al., 2013; Wickramarachchi et al., 2008)	Yes
PF3D7_0323400	Ripr (RH5 interacting protein)	D21—N1086 (1065, N1086)	4	No	BAP-Strep	Cannot be knocked out in *P. falciparum* (Chen et al., 2011)	Yes
PF3D7_0828800	GAMA (GPI-anchored micronemal antigen)	R24—S716 (692, N738)	6	Yes	BAP-Strep	Susceptible to vaccine-induced antibody *in vitro* (Arumugam et al., 2011)	Yes
PF3D7_0207600	SERA5 (serine-rich repeat antigen 5)	T23—V997 (974, V997)	9	Yes	BAP-Strep	Susceptible to vaccine-induced antibody *in vivo* (Inselburg et al., 1991)	No: not in Hu et al., (2010) list of 435 invasion-associated genes

PF3D7_1033200	Early transcribed membrane protein 10.2 (ETRAMP)	M77—E355 (278, E355)	3	No	BAP-Strep	Homologue cannot be knocked out in *P. vivax* (MacKellar et al., 2011)	No: not in Hu et al., (2010) list of 435 invasion-associated genes
PF3D7_1035200	S-antigen	K20—M585 (565, M585)	NC	Yes	BAP-Strep	Cannot be knocked out (Cowman and Crabb, 2006; Cowman et al., 2000)	No: not in Hu et al., (2010) list of 435 invasion-associated genes
PF3D7_0707300	RAMA (rhoptry-associated membrane antigen)	L18—S841 (823, N861)	7	No	BAP-Strep	Cannot be knocked out (Sanders et al., 2006), sero-epidemiological association with protection (Nixon et al., 2005)	Yes
PF3D7_0905400	RhopH3 (high molecular weight rhoptry protein 3)	G24—L897 (873, L897)	3	No	BAP-Strep	Reportedly cannot be knocked out (Cowman and Crabb, 2006)	Yes
PF3D7_1364100	Pf92	S16—D768 (752, I796)	4	Yes	BAP-Strep	Reportedly cannot be knocked out (Cowman and Crabb, 2006)	No: not in Hu et al., (2010) list of 435 invasion-associated genes
PF3D7_1035400	MSP3	K26—H354 (328, H354)	7	No	BAP-Strep	Susceptible to vaccine-induced antibodies in humans (Sirima et al., 2011)	Yes
PF3D7_0207000	MSP4	Y29—S248 (219, S248)	5	No	BAP-Strep	Susceptible to vaccine-induced antibodies *in vitro* (de Silva et al., 2011)	Yes

PF3D7_0423800	CyRPA (native)	D29—E362 (333, E362)	1	Yes	BAP-6His	Susceptible to vaccine-induced antibodies *in vitro* (Dreyer et al., 2012; Reddy et al., 2015)	Yes
PF3D7_0423800	CyRPA (Gly KO)	D29—E362 (333, E362), plus point mutations S147A, T324A and T340A	As above	Yes	See Figure S4 and (Bushell et al., 2008) for construct details	Susceptible to vaccine-induced antibodies *in vitro* (Dreyer et al., 2012; Reddy et al., 2015)	Yes
PF3D7_1021800	SEA1 (native)	R810—E1083 (273, I2074)	0	Yes	BAP-Strep	Susceptible to vaccine-induced antibodies *in vitro* and *in vivo* (Raj et al., 2014)	No: not in Hu et al., (2010) list of 435 invasion-associated genes
PF3D7_1021800	SEA1 (Gly KO)	R810—E1083 (273, I2074), plus point mutations N1022Q and N1069Q	0	Yes	BAP-Strep	Susceptible to vaccine-induced antibodies *in vitro* and *in vivo* (Raj et al., 2014)	No: not in Hu et al., (2010) list of 435 invasion-associated genes

[a] Shown as: First AA – Last AA (length of construct, last AA in protein).

Table S2: Proteins with bioinformatically-predicted role in RBC invasion (N=40, see also Fig S2)

Gene	Protein	Construct[a]	SNPs	Vaccine obtained?	C-terminal tags
PF3D7_1028700	MTRAP (merozoite-specific thrombospondin-related anonymous protein)	I23—K432 (409, E498)	1	Yes	BAP-6His
PF3D7_1035700	DBLMSP (Duffy binding like Mz surface protein)	K24—N457 (433, K697)	25	Yes	BAP-6His
PF3D7_0405900	ASP (apical sushi protein)	K21—K711 (690, A731)	2	Yes	BAP-6His
PF3D7_1367900	Hypothetical protein, conserved	R26—I647 (621, I647)	0	Yes	BAP-6His
PF3D7_0404700	DPAP3 (dipeptidyl peptidase 3)	D21—T939 (918, T939)	13	No	BAP-6His
PF3D7_1030200	Hypothetical protein (had TM)	M40—S138 (98, F450)	4	No	BAP-6His
PF3D7_0207500	SERA6 (serine repeat antigen 6)	K25—V1031 (1006, V1031)	1	No	BAP-6His
PF3D7_0207800	SERA3 (serine repeat antigen 3)	T23—I930 (907, I930)	1	No	BAP-6His
PF3D7_0208000	SERA1 (serine repeat antigen 1)	E25—V994 (969, V994)	1	No	BAP-6His
PF3D7_1035300	GLURP (glutamate-rich protein)	K24—I1233 (1209, I1233)	16	No	BAP-6His
PF3D7_1035100	hypothetical protein	N26—K561 (535, K561)	23	No	BAP-6His
PF3D7_1035900	hypothetical protein	A21—P566 (545, P566)	5	No	BAP-6His
PF3D7_1102300	hypothetical protein	P32—V415 (383, V415)	2	No	BAP-6His

PF3D7_1116000	RON4 (rhoptry neck protein 4)	F24—L1201 (1177, L1201)	1	No	BAP-6His
PF3D7_1136200	hypothetical protein	I21—L679 (658, L679)	1	No	BAP-6His
PF3D7_1404700	hypothetical protein	Q21—K290 (269, K290)	3	Yes	BAP-6His
PF3D7_1463900	hypothetical protein	E23—N445 (422, N445)	5	No	BAP-6His
PF3D7_1321900	hypothetical protein, conserved	K21—S292 (271, S292)	2	Yes	BAP-6His
PF3D7_1036000	MSP11 (AKA MSP3.7 AKA H103)	S25—Y405 (380, Y405)	1	Yes	BAP-6His
PF3D7_0722200	RALP1 (rhoptry-associated leucine zipper-like protein 1)	S23—F749 (726, F749)	0	No	BAP-6His
PF3D7_0815300	FAD-dependent monooxygenase (putative)	K26—I1182 (1156, I1182)	7	No	BAP-6His
PF3D7_1143200	DnaJ protein (putative)	L22—M109 (87, D321)	1	No	BAP-6His
PF3D7_0404800	hypothetical protein, conserved	L24—E567 (543, E567)	3	No	BAP-6His
PF3D7_0423300	hypothetical protein, conserved	E42—R476 (434, R476)	2	No	BAP-6His
PF3D7_0507300	hypothetical protein, conserved	Y19—R893 (874, R893)	4	No	BAP-6His
PF3D7_0932100	hypothetical protein, conserved	K143—S1274 (1131, S1274)	5	No	BAP-6His
PF3D7_1216500	MDV1 (male development gene 1)	F20—D221 (201, D221)	0	No	BAP-6His
PF3D7_1229300	hypothetical protein, conserved	L20—S890 (870, A990)	8	No	BAP-6His

PF3D7_1246000	hypothetical protein, conserved	K25—L443 (418, L483)	2	No	BAP-6His
PF3D7_0214900	RON6 (rhoptry neck protein 6)	F16—G950 (934, G950)	3	Yes	BAP-6His
PF3D7_0620400	MSP10 (merozoite surface protein 10)	H27—K506 (479, K506)	7	Yes	BAP-6His
PF3D7_0629300	phosphatidylcholine-sterol acyltransferase (putative)	G21—N863 (842, N863)	13	No	BAP-6His
PF3D7_0507500	SUB1 (subtilisin-like protease 1)	E31—H688 (657, H688)	3	No	BAP-6His
PF3D7_1335100	MSP7 (merozoite surface protein 7)	N22—M351 (329, M351)	13	Yes	BAP-6His
PF3D7_0935800	RhopH1 (high molecular weight rhoptry protein 1)	L268—F1340 (1072, F1340)	8	No	BAP-6His
PF3D7_0808200	Plasmepsin X	C26—N573 (547, N573)	4	Yes	BAP-6His
PF3D7_0929400	Rhop H2 (high molecular weight rhoptry protein 2)	K25—S1378 (1353, S1378)	6	No	BAP-6His
PF3D7_0220800	RhopH1 (high molecular weight rhoptry protein 1)	S25—Q1440 (1415, Q1440)	16	No	BAP-6His
PF3D7_1322000	adenosine-diphosphatase	Q31—L565 (534, L565)	5	No	BAP-6His
PF3D7_0831600	CLAG8 (Cytoadherence linked asexual protein 8)	S25—E1394 (1369, E1394)	>20	No	BAP-6His

[a] Shown as: First AA – Last AA (length of construct, last AA in protein).

Table S3: Staining pattern of anti-merozoite antibody specificities against late-stage schizonts

**Protein**	**Staining pattern**	**As expected?**	**Comment**
SEA-1 (Gly KO)	Diffuse, non-uniform	Yes	As observed by Raj et al. (2014).
SEA-1 (native)	As above, but higher background	Yes	As above, but high background suggestive of more non-specific binding.
P113	Surface edge of schizont	Unclear	Our findings are consistent with proteomics work (Elsworth et al., 2016) but this protein has also been described as an apical merozoite protein (Galaway et al., 2017).
CyRPA (Gly KO)	Sharply punctate,	Yes	As seen by Volz et al. (2016).
CyRPA (native)	Punctate, less sharp, very bright	Yes	As above, but less localization suggests non-specific binding.
PTRAMP	Usual punctate/merozoite surface combination	Yes	Thompson et al. (2004) saw PTRAMP in a punctate pattern in early schizonts, moving to a merozoite surface pattern in segmenting schizonts.
AARP	Blank	No	See section 3.4 of main text.
GAMA	Punctate	Yes	Seen in micronemes by (Arumugam et al., 2011).
SERA5	Merozoite surface	Yes	As seen by Li et al. (2002).
S-antigen	Punctate through schizont, also surface edge of schizont	NA	Not yet localized.
Pf3D7_1404700	Punctate	NA	Not yet localized.
MTRAP	Punctate	Yes	As seen by Riglar et al. (2016).
DBLMSP	Merozoite surface	Yes	As seen by Crosnier et al. (2016)
ASP	Similar to S-antigen: punctate through schizont, also surface edge of schizont	Unclear	Previous studies have observed a punctate pattern (Srivastava et al., 2010; Zuccala et al., 2012), but the surface staining seen in this study has not been observed before.
PF3D7_1367900	Merozoite surface	NA	Not yet localized.
Pf92	Punctate	Unclear	A previous study localized Pf92 to the merozoire surface in schizont, however, this was a GFP-tagged construct that may have had altered trafficking (Sanders et al., 2005).
PF3D7_1321900	Diffuse with bright, punctate areas	NA	Not yet localized.

MSP11	Blank	No	Inoculum was probably non-immunogenic
RON6	Punctate	Yes	As seen by Proellocks et al. (2009)
MSP10	Merozoite surface	Yes	As seen by Maskus et al. (2015)
MSP7	Merozoite surface	Yes	As seen by Kadekoppala et al. (2008)
Plasmepsin X	Punctate, speckly	Yes	Shown to localize to exonemes by EM in Nasamu et al. (2017); IFA image of exonemes in Yeoh et al. (2007)

Supplementary Material References

Arumugam, T.U., Takeo, S., Yamasaki, T., Thonkukiatkul, A., Miura, K., Otsuki, H., Zhou, H., Long, C.A., Sattabongkot, J., Thompson, J., et al. (2011). Discovery of GAMA, a Plasmodium falciparum merozoite micronemal protein, as a novel blood-stage vaccine candidate antigen. Infect. Immun. *79*, 4523–4532.

Bushell, K.M., Sollner, C., Schuster-Boeckler, B., Bateman, A., and Wright, G.J. (2008). Large-scale screening for novel low-affinity extracellular protein interactions. Genome Res. *18*, 622–630.

Chen, L., Lopaticki, S., Riglar, D.T., Dekiwadia, C., Uboldi, A.D., Tham, W.H., O'Neill, M.T., Richard, D., Baum, J., Ralph, S.A., et al. (2011). An EGF-like protein forms a complex with PfRh5 and is required for invasion of human erythrocytes by Plasmodium falciparum. PLoS Pathog. *7*, e1002199.

Cowman, A.F., and Crabb, B.S. (2006). Invasion of red blood cells by malaria parasites. Cell *124*, 755–766.

Cowman, A.F., Baldi, D.L., Healer, J., Mills, K.E., O'Donnell, R.A., Reed, M.B., Triglia, T., Wickham, M.E., and Crabb, B.S. (2000). Functional analysis of proteins involved in Plasmodium falciparum merozoite invasion of red blood cells. FEBS Lett. *476*, 84–88.

Crosnier, C., Iqbal, Z., Knuepfer, E., Maciuca, S., Perrin, A.J., Kamuyu, G., Goulding, D., Bustamante, L.Y., Miles, A., Moore, S.C., et al. (2016). Binding of Plasmodium falciparum Merozoite Surface Proteins DBLMSP and DBLMSP2 to Human Immunoglobulin M Is Conserved among Broadly Diverged Sequence Variants. J. Biol. Chem. *291*, 14285–14299.

Dreyer, A.M., Matile, H., Papastogiannidis, P., Kamber, J., Favuzza, P., Voss, T.S., Wittlin, S., and Pluschke, G. (2012). Passive immunoprotection of Plasmodium falciparum-infected mice designates the CyRPA as candidate malaria vaccine antigen. J. Immunol. *188*, 6225–6237.

Edgar, R.C. (2004). MUSCLE: multiple sequence alignment with high accuracy and high throughput. Nucleic Acids Res. *32*, 1792–1797.

Elsworth, B., Sanders, P.R., Nebl, T., Batinovic, S., Kalanon, M., Nie, C.Q., Charnaud, S.C., Bullen, H.E., de Koning Ward, T.F., Tilley, L., et al. (2016). Proteomic analysis reveals novel proteins associated with the Plasmodium protein exporter PTEX and a loss of complex stability upon truncation of the core PTEX component, PTEX150. Cell. Microbiol. *18*, 1551–1569.

Galaway, F., Drought, L.G., Fala, M., Cross, N., Kemp, A.C., Rayner, J.C., and Wright, G.J. (2017). P113 is a merozoite surface protein that binds the N terminus of Plasmodium falciparum RH5. Nat. Commun. *8*, 14333.

Hu, G., Cabrera, A., Kono, M., Mok, S., Chaal, B.K., Haase, S., Engelberg, K., Cheemadan, S., Spielmann, T., Preiser, P.R., et al. (2010). Transcriptional profiling of growth perturbations of the human malaria parasite Plasmodium falciparum. Nat. Biotechnol. *28*, 91–98.

Inselburg, J., Bzik, D.J., Li, W.B., Green, K.M., Kansopon, J., Hahm, B.K., Bathurst, I.C., Barr, P.J., and Rossan, R.N. (1991). Protective immunity induced in Aotus monkeys by recombinant SERA proteins of Plasmodium falciparum. Infect. Immun. *59*, 1247–1250.

Kadekoppala, M., O'Donnell, R.A., Grainger, M., Crabb, B.S., and Holder, A.A. (2008). Deletion of the Plasmodium falciparum merozoite surface protein 7 gene impairs parasite invasion of erythrocytes. Eukaryot. Cell *7*, 2123–2132.

Li, J., Mitamura, T., Fox, B.A., Bzik, D.J., and Horii, T. (2002). Differential localization of processed fragments of Plasmodium falciparum serine repeat antigen and further processing of its N-terminal 47 kDa fragment. Parasitol. Int. *51*, 343–352.

MacKellar, D.C., Vaughan, A.M., Aly, A.S.I., DeLeon, S., and Kappe, S.H.I. (2011). A systematic analysis of the early transcribed membrane protein family throughout the life cycle of Plasmodium yoelii. Cell. Microbiol. *13*, 1755–1767.

Maskus, D.J., Bethke, S., Seidel, M., Kapelski, S., Addai-Mensah, O., Boes, A., Edgü, G., Spiegel, H., Reimann, A., Fischer, R., et al. (2015). Isolation, production and characterization of fully human monoclonal antibodies directed to Plasmodium falciparum MSP10. Malar. J. *14*, 276.

Nasamu, A.S., Glushakova, S., Russo, I., Vaupel, B., Oksman, A., Kim, A.S., Fremont, D.H., Tolia, N., Beck, J.R., Meyers, M.J., et al. (2017). Plasmepsins IX and X are essential and druggable mediators of malaria parasite egress and invasion. Science (80-.). *358*, 518–522.

Nixon, C.P., Friedman, J., Treanor, K., Knopf, P.M., Duffy, P.E., and Kurtis, J.D. (2005). Antibodies to rhoptry-associated membrane antigen predict resistance to Plasmodium falciparum. J. Infect. Dis. *192*, 861–869.

Pandey, A.K., Reddy, K.S., Sahar, T., Gupta, S., Singh, H., Reddy, E.J., Asad, M., Siddiqui, F.A., Gupta, P., Singh, B., et al. (2013). Identification of a potent combination of key Plasmodium falciparum merozoite antigens that elicit strain-transcending parasite-neutralizing antibodies. Infect. Immun. *81*, 441–451.

Proellocks, N.I., Kats, L.M., Sheffield, D.A., Hanssen, E., Black, C.G., Waller, K.L., and Coppel, R.L. (2009). Characterisation of PfRON6, a Plasmodium falciparum rhoptry neck protein with a novel cysteine-rich domain. Int. J. Parasitol. *39*, 683–692.

Raj, D.K., Nixon, C.P., Nixon, C.E., Dvorin, J.D., DiPetrillo, C.G., Pond-Tor, S., Wu, H.-W., Jolly, G., Pischel, L., Lu, A., et al. (2014). Antibodies to PfSEA-1 block parasite egress from RBCs and protect against malaria infection. Science (80-.). *344*, 871–877.

Reddy, K.S., Amlabu, E., Pandey, A.K., Mitra, P., Chauhan, V.S., and Gaur, D. (2015). Multiprotein

complex between the GPI-anchored CyRPA with PfRH5 and PfRipr is crucial for *Plasmodium falciparum* erythrocyte invasion. Proc. Natl. Acad. Sci. *112*, 1179–1184.

Riglar, D.T., Whitehead, L., Cowman, A.F., Rogers, K.L., and Baum, J. (2016). Localisation-based imaging of malarial antigens during erythrocyte entry reaffirms a role for AMA1 but not MTRAP in invasion. J. Cell Sci. *129*, 228–242.

Sanders, P.R., Gilson, P.R., Cantin, G.T., Greenbaum, D.C., Nebl, T., Carucci, D.J., McConville, M.J., Schofield, L., Hodder, A.N., Yates, J.R., et al. (2005). Distinct Protein Classes Including Novel Merozoite Surface Antigens in Raft-like Membranes of *Plasmodium falciparum*. J. Biol. Chem. *280*, 40169–40176.

Sanders, P.R., Kats, L.M., Drew, D.R., O'Donnell, R.A., O'Neill, M., Maier, A.G., Coppel, R.L., and Crabb, B.S. (2006). A set of glycosylphosphatidyl inositol-anchored membrane proteins of Plasmodium falciparum is refractory to genetic deletion. Infect. Immun. *74*, 4330–4338.

de Silva, H.D., Saleh, S., Kovacevic, S., Wang, L., Black, C.G., Plebanski, M., and Coppel, R.L. (2011). The antibody response to Plasmodium falciparum Merozoite Surface Protein 4: comparative assessment of specificity and growth inhibitory antibody activity to infection-acquired and immunization-induced epitopes. Malar. J. *10*, 266.

Sirima, S.B., Cousens, S., and Druilhe, P. (2011). Protection against malaria by MSP3 candidate vaccine. N. Engl. J. Med. *365*, 1062–1064.

Srivastava, A., Singh, S., Dhawan, S., Mahmood Alam, M., Mohmmed, A., and Chitnis, C.E. (2010). Localization of apical sushi protein in Plasmodium falciparum merozoites. Mol. Biochem. Parasitol. *174*, 66–69.

Thompson, J., Cooke, R.E., Moore, S., Anderson, L.F., Janse, C.J., and Waters, A.P. (2004). PTRAMP; a conserved Plasmodium thrombospondin-related apical merozoite protein. Mol. Biochem. Parasitol. *134*, 225–232.

Volz, J.C., Yap, A., Sisquella, X., Thompson, J.K., Lim, N.T.Y., Whitehead, L.W., Chen, L., Lampe, M., Tham, W.-H., Wilson, D., et al. (2016). Essential Role of the PfRh5/PfRipr/CyRPA Complex during Plasmodium falciparum Invasion of Erythrocytes. Cell Host Microbe *20*, 60–71.

Wickramarachchi, T., Devi, Y.S., Mohmmed, A., and Chauhan, V.S. (2008). Identification and characterization of a novel Plasmodium falciparum merozoite apical protein involved in erythrocyte binding and invasion. PLoS One *3*, e1732.

Williams, A.R., Douglas, A.D., Miura, K., Illingworth, J.J., Choudhary, P., Murungi, L.M., Furze, J.M., Diouf, A., Miotto, O., Crosnier, C., et al. (2012). Enhancing blockade of Plasmodium falciparum erythrocyte invasion: assessing combinations of antibodies against PfRH5 and other merozoite antigens. PLoS Pathog *8*, e1002991.

Yeoh, S., O'Donnell, R.A., Koussis, K., Dluzewski, A.R., Ansell, K.H., Osborne, S.A., Hackett, F., Withers-Martinez, C., Mitchell, G.H., Bannister, L.H., et al. (2007). Subcellular discharge of a serine protease mediates release of invasive malaria parasites from host erythrocytes. Cell *131*, 1072–1083.

Zuccala, E.S., Gout, A.M., Dekiwadia, C., Marapana, D.S., Angrisano, F., Turnbull, L., Riglar, D.T.,

Rogers, K.L., Whitchurch, C.B., Ralph, S.A., et al. (2012). Subcompartmentalisation of proteins in the rhoptries correlates with ordered events of erythrocyte invasion by the blood stage malaria parasite. PLoS One *7*, e46160.
